# Estimating causal effects of time-dependent exposures on a binary endpoint in a high-dimensional setting

**DOI:** 10.1186/s12874-018-0527-5

**Published:** 2018-07-03

**Authors:** Vahé Asvatourian, Clélia Coutzac, Nathalie Chaput, Caroline Robert, Stefan Michiels, Emilie Lanoy

**Affiliations:** 1 Université Paris-Saclay, Univ. Paris-Sud, UVSQ, CESP, INSERM, Villejuif, France; 2 Service de Biostatistique et d’Epidémiologie, Gustave-Roussy, Villejuif, France; 3 Gustave Roussy, Laboratoire d’Immunomonitoring en Oncologie, and CNRS-UMS 3655 and INSERM-US23, F-94805 Villejuif, France; 4 Université Paris-Sud, Faculté de Médecine, F-94276 Le Kremlin Bicêtre, France; 5 Université Paris-Sud, Faculté de Pharmacie, F-92296 Chatenay-Malabry, France; 6 Department of Medicine, Dermatology Unit, Gustave Roussy Cancer Campus, Villejuif, France

**Keywords:** Repeated measures, Immunotherapy, PC-algorithm, IDA, High dimensional setting, Causal inference, Observational data

## Abstract

**Background:**

Recently, the intervention calculus when the DAG is absent (IDA) method was developed to estimate lower bounds of causal effects from observational high-dimensional data. Originally it was introduced to assess the effect of baseline biomarkers which do not vary over time. However, in many clinical settings, measurements of biomarkers are repeated at fixed time points during treatment and, therefore, this method needs to be extended. The purpose of this paper is to extend the first step of the IDA, the Peter Clarks (PC)-algorithm, to a time-dependent exposure in the context of a binary outcome.

**Methods:**

We generalised the so-called “PC-algorithm” to take into account the chronological order of repeated measurements of the exposure and proposed to apply the IDA with our new version, the chronologically ordered PC-algorithm (COPC-algorithm). The extension includes Firth’s correction. A simulation study has been performed before applying the method for estimating causal effects of time-dependent immunological biomarkers on toxicity, death and progression in patients with metastatic melanoma.

**Results:**

The simulation study showed that the completed partially directed acyclic graphs (CPDAGs) obtained using COPC-algorithm were structurally closer to the true CPDAG than CPDAGs obtained using PC-algorithm. Also, causal effects were more accurate when they were estimated based on CPDAGs obtained using COPC-algorithm. Moreover, CPDAGs obtained by COPC-algorithm allowed removing non-chronological arrows with a variable measured at a time *t* pointing to a variable measured at a time t´ where *t´* < *t*. Bidirected edges were less present in CPDAGs obtained with the COPC-algorithm, supporting the fact that there was less variability in causal effects estimated from these CPDAGs. In the example, a threshold of the per-comparison error rate of 0.5% led to the selection of an interpretable set of biomarkers.

**Conclusions:**

The COPC-algorithm provided CPDAGs that keep the chronological structure present in the data and thus allowed to estimate lower bounds of the causal effect of time-dependent immunological biomarkers on early toxicity, premature death and progression.

**Electronic supplementary material:**

The online version of this article (10.1186/s12874-018-0527-5) contains supplementary material, which is available to authorized users.

## Background

The Intervention calculus when the directed acyclic graph (DAG) is absent (IDA) method was recently developed to estimate lower bound of total causal effects from observational data in high-dimensional settings [1]. It was originally introduced to evaluate the effect of time-fixed exposure (gene expression). This method is a combination of Peter Clarks (PC)-algorithm [2] and Pearl’s do calculus [3]. The PC-algorithm is a constraint-based method for causal structure learning, meaning that it learns the causal structure based on the conditional dependencies of the observational distribution. The output of the PC-algorithm results in a CPDAG (completed partially DAG) that encodes conditional dependencies of the data in a class of DAGs (Directed acyclic graphs) called *Markov Equivalent*. Then, based on the DAGs in the *Markov Equivalence Class*, causal effects are estimated using Pearl’s do calculus (see section Causal effect estimation in high dimensional settings). However, in many clinical settings, time-dependent biomarker values under treatment or changes in biomarkers from baseline are of interest. If the true DAG was known, the commonly used marginal structural model (MSM) approach could be used to estimate causal effects in the case of time-dependent covariates and outcome [4, 5]. In our setting, the true DAG being unknown, causal effects could not be identified using MSM.

In the 2010s, new anti-cancer treatments targeting immune checkpoints were introduced: the wave of these immunotherapies began with the anti CTLA-4 treatment which showed a survival benefit in patients with metastatic melanoma [6, 7]. More recently, promising results in lung and kidney cancers have also been obtained [8]. Nevertheless, only a subgroup of patients seem to benefit from this treatment: about 20% of patients with metastatic melanoma treated with ipilimumab were long-term survivors (3 years) [9]. Moreover, immune-related toxicity such as colitis occurs in 8 to 22% of treated patients [10]. The goal of immunotherapy is to amplify the immune system response against cancer cells. Thus, one can observe the evolution of the treatment by looking at the immune system. Predictive and/or prognostic markers are ideally validated through clinical trials including randomized studies, which are the gold standard [11, 12]. Before being evaluated in randomized trial, candidate immunological biomarkers can be identified from high-dimensional data, collected in an observational or non-randomized setting.

Our objective was to develop methods to identify the causal effects of time-dependent exposures on a binary endpoint in a high-dimensional setting, with an application of time-dependent immunological biomarkers in a non-randomized prospective study in oncology. However, the PC-algorithm has never been applied on data measured repeatedly at a fixed time points, and the chronological order among data is not respected when using PC-algorithm. The first step was then to find the true CPDAG by extending the PC-algorithm to chronologically ordered measures and then to estimate robust causal effects based on the CPDAG estimated using our version of the PC-algorithm. To ensure the accuracy and the efficiency of our method, we made a simulation study where we compared the CPDAGs’ structure obtained using PC-algorithm and our method. Then we compared the estimation of true causal effects calculated based on CPDAGs obtained from both methods. Due to collinearity among time-dependent biomarkers, we added for the first time the Firth’s correction while estimating causal effects to avoid instability of the maximum likelihood estimates. After the simulation study, we applied both PC-algorithm and our method to real dataset of time-dependent immunological biomarkers.

## Methods

### Graph definitions and notations

Let *G* (*N*, *E*) be a graph consisting of nodes *N* and edges *E*. Nodes represent random variables *N* = {*X*_1_, …, *X*_*p*_} and edges represent the links between them. An edge can be either directed *X*_*i*_ → *X*_*j*_ (in this case, *X*_*i*_ is a parent of *X*_*j*_ and *X*_*j*_ is a descendant of *X*_*i*_) or undirected *X*_*i*_ − *X*_*j*._ A graph with only undirected edges is said to be an undirected graph whereas a directed graph is made of only directed edges. A partially directed graph contains both directed and undirected edges.

Two nodes are said to be adjacent if they are connected by an edge (either directed or undirected). A path is a sequence of nodes in which all pairs are adjacent. A path can be either *open* or *closed*. A path is open when there is no collision between two arrows pointing to the same node on the path (i.e. the path from *X*_*i*_ to *X*_*m*_ in (1) is open).

1 $$ {X}_i\to {X}_j\to {X}_k\to {X}_m\leftarrow {X}_l $$

A path is closed when there is a collision between two arrows which point to the same node of the path, this variable is a collider (i.e, the path from *X*_*i*_ to *X*_*l*_ in (1) is closed). We denote *X*_*k*_ as a descendent of *X*_*i*_ (and *X*_*i*_ an ancestor of *X*_*k*_) if there is a path that starts from *X*_*i*_ and ends to *X*_*k*_ by following the direction of the arrows (1). We also denote *pa*(*X*_*i*_, *G*) as the parents of *X*_*i*_ in *G* by the set of variables pointing to *X*_*i*_. A graph is called acyclic when no path starts and ends at the same node. A graph which is acyclic and has directed edges is called a directed acyclic graph (DAG). A DAG is complete or statistical when all pairs of nodes are adjacent, whereas a DAG is causal when all common causes of any variable are on the graph, i.e. any parent is a cause of its descendants. Therefore, a causal DAG is informative whereas a complete DAG is non-informative because a lack of arrow means an absence of a direct causal effect.

A graph encodes (conditional) independence relationships through the concept of *d-separation* [13]. If two nodes are d-separated by a set of nodes, then the variables corresponding to the nodes are conditionally independent given this set of variables. The set of these given variables is then called separation set ***S***. Multiple DAGs can be compatible with a same set of underlying conditional independences. Let a skeleton be the graph obtained by removing all arrowheads from the DAG and the *v-structures* a subgraph of 3 nodes filling two conditions: 1) both arrows are not pointed on *X*_*j*_ (*X*_*j*_ is not a *collider*) and 2) where *X*_*i*_ and *X*_*k*_ are not adjacent.

The DAGs X_*i*_ → *X*_*j*_ → *X*_*k*_, X_*i*_ ← *X*_*j*_ ← *X*_*k*_ and X_*i*_ ← *X*_*j*_ → *X*_*k*_ in which the two conditions hold, belong to the same *Markov equivalence class* and are called *Markov equivalent*. A whole equivalence class can be summarised in a graph that has the same skeleton and includes the directed arrows of all DAGs in the equivalence class. Edges which are directed differently across the DAGs in the equivalence class are represented with bidirected arrows (or simply edges). This graph with both undirected and directed edges is called a *Completed Partially DAG* (CPDAG).

### Causal effect estimation in high dimensional settings

#### IDA

**Fig. 1:**
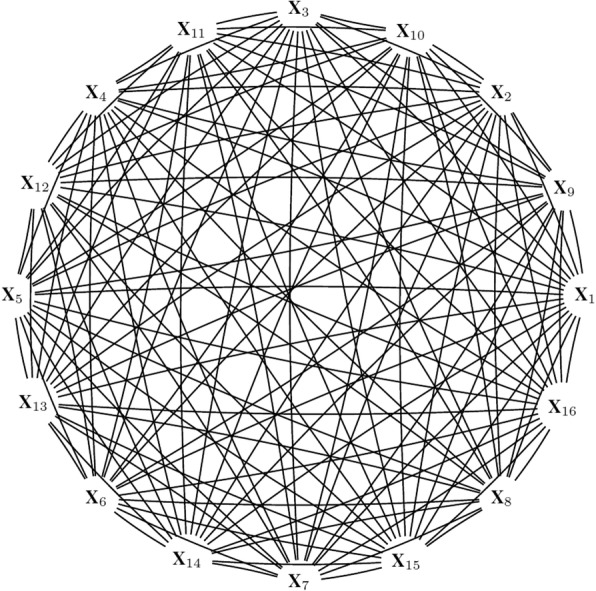
A complete undirected graph

When the relationships between variables are not oriented, the DAG cannot be identified. With many variables in a high-dimensional setting, it is not possible to determine which nodes are ancestors and which are descendants. The only possible initial graph that can be drawn based on high-dimensional data is a complete undirected graph which is non-informative (Fig. 1). The intervention calculus when the DAG is absent (IDA) method has been introduced to determine the CPDAG from the observational data and to estimate lower bounds of the absolute values of the total causal effects in the case where all variables (including outcome) are continuous [1]; and has been extended to the case where all variables are binary [14]. The first objective of the IDA is to estimate the CPDAG and its Markov equivalence class that contain the true causal DAG from the observational data by using a causal learning algorithm such as the PC-algorithm [2]. Then the intervention calculus [3, 15] is used on the *m* DAGs_*j*_ of the Markov equivalence class *j* = 1, …, *m*, to estimate the *p* × *m* matrix *Θ* of causal effects *θ*_*ij*_ of each covariate *X*_*i*_ (*i* = 1, …, *p*) on *Y*.

However, estimating the true causal effect is impossible when a unique DAG is not identifiable. To determine whether or not a covariate has a potential causal effect, the minimum absolute causal effect of a covariate is defined as$\widehat{\kern0.5em {\beta}_i}={\min}_j\left(|{\widehat{\theta}}_{ij}|\right)$. Then a ranking of covariates’ causal effects is made based on these lower bounds, where ${\widehat{\beta}}_{i_1}$ is the lower bound of the covariate *i* with the rank 1:

2 $$ {\widehat{\beta}}_{i_1}\ge {\widehat{\beta}}_{i_2}\ge \dots \ge {\widehat{\beta}}_{i_p}. $$

Determining all the DAGs that are present in the Markov equivalence class can be highly computationally intensive in a high-dimensional setting. Nevertheless, rather than computing all the DAGs, it is still possible to determine the set of parents used for adjusting by extracting them from the CPDAG. The local algorithm used by Maathuis et al. [1] checks if the parents are locally valid (if they create or not a new collider) in the CPDAG and all causal estimates for a single covariate *X*_*i*_ on *Y* are in the multiset *Θ*_*i*_ = {*θ*_*ij*_}_*j* ∈ {1, …, *m*} and *i* ∈ {1, …, *p*}_. Contrary to a set, in a multiset the replication of an element matters. For instance, the multisets {*a*, *a*, *b*} and {*a*, *b*} are not equal while the sets {*a*, *a*, *b*} and {*a*, *b*} are. The multiset allows the multiplicity of an element.

Finally, the assumptions made in the IDA are:

(A1) There are no hidden variables.

(A2) The joint distribution of covariates *X*_*i*_, …, *X*_*p*_ is normal and faithful to the true (unknown) DAG.

(A3) Covariates *X*_*i*_, …, *X*_*p*_ have equal variance_._

The IDA method developed by Maathuis et al. is implemented in the R-package pcalg [16].

#### PC-algorithm

The PC-algorithm is a constraint based method for causal structure learning [2, 17], meaning that it learns the causal structure based on the conditional dependencies between variables. A sketch of the PC-algorithm is given in algorithm 1.

First, it estimates the skeleton of the underlying structure by checking all given conditional dependencies between each variable at a significance level α. If no information on dependencies is given, then the graph used as input is an *undirected graph* such as in Fig. 1. Once the skeleton is obtained, edges are oriented in the *v-structures* to meet the conditional dependencies and finally the CPDAG is obtained by directing as many remaining edges as possible according to three rules (see section 3.1 of [18]):

R1: When there is a directed edge *X*_*i*_ → *X*_*j*_, *X*_*j*_ − *X*_*k*_ is oriented into *X*_*j*_ → *X*_*k*_ with *X*_*i*_ and *X*_*k*_ not adjacent;

R2: When there is a chain *X*_*i*_ → *X*_*k*_ → *X*_*j*_,  *X*_*i*_ − *X*_*j*_ is oriented into *X*_*i*_ → *X*_*j*_;

R3: When there are two chains *X*_*i*_ − *X*_*l*_ → *X*_*j*_ and *X*_*i*_ − *X*_*k*_ → *X*_*j*_,  *X*_*i*_ − *X*_*j*_ is oriented into *X*_*i*_ → *X*_*j* _with *X*_*k*_ and *X*_*l*_ not adjacent.

Even though the PC-algorithm has been shown to be consistent in high-dimensional settings [19], one of its issues remains the effect of the set of ordered variables ***O*** in the final output. In fact, the order of the variables determines which pair of nodes is tested first, determining which edges are removed first and so affecting which tests are considered later on. This order dependence impacts robustness of the results in high-dimensional settings. Two different solutions have been suggested: the stability ranking and the PC-stable, which will be outlined below. Before running the algorithm, the multiple testing requires to specify the significance level (cut-off) α for the conditional independence tests. In fact, setting α to a certain value means that only conditional dependencies with a *p*-value under α are kept. Thus, running PC-algorithm with a small value of alpha leads to obtaining sparser graphs.

#### Stability selection

To deal with the order dependence issue of the PC-algorithm in the IDA which can lead to poor robustness, Stekhoven et al. proposed to add a stability selection step [20] to IDA. This method, called Causal Stability Ranking (CStaR) [21], is based on a re-sampling approach. The IDA is run over 100 independent random subsamples and then in each subsampling run, the variables are ranked according to Eq. (2). At the end of all runs, the relative frequencies *Π*_*i*_ of covariates appearing among the top of *q* variables are used to define a stable ranking:

3 $$ {\widehat{\Pi}}_1\ge {\widehat{\Pi}}_2\ge \dots \ge {\widehat{\Pi}}_p.\kern0.5em $$

For a given *q*, a bound for the per-comparison error rate (PCER), which can be seen as the false positive error rate, is given by:

4 $$ \frac{1}{2\hat{\Pi_j}-1}\frac{q^2}{p^2}.\kern1.5em $$

#### PC-stable

Another approach that considers the order dependence issue of the PC-algorithm was explored by Colombo and Maathuis by introducing an order-independent version of the PC-algorithm called PC-stable [18]. In step 1 of the PC-stable version, the adjacency set of all variables are stored after each change in the size of the separation set (see section “Discussion”.1 of [18]); removing an edge will not affect which conditional independencies are checked for other pairs of variables. In addition, they also showed that the combination of the stability selection with PC-stable gave more reliable edges than PC-stable alone on yeast gene expression data [18].

#### Extension to a time-dependent exposure

We aimed to extend the IDA by integrating time-dependent exposures in the PC-stable step. Based on chronologically ordered data, the resulting CPDAG should not contain arrow from a descendant to a parent *X*_1, *t*′_ → *X*_1, *t*_ where *t* < *t'* since the value of a variable at time *t'* cannot influence a past value of the same variable. This means also that in the first step, when looking at conditional dependencies between two variables measured at time *t* and *t*^∗^ where *t* ≥ *t*^∗^, variables measured at a time *t'* where *t'* > *t* and *t'* > *t*^∗^ should not be tested for the separation set ***S***.

**Fig. 2:**
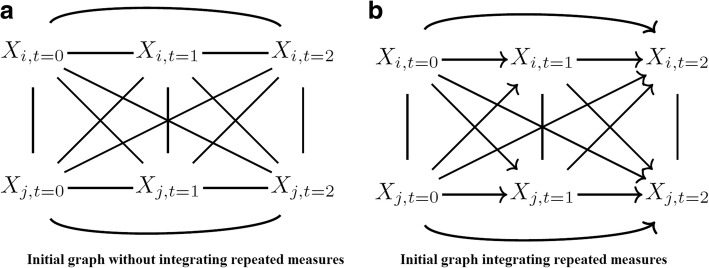
Initial graphs used as input for the IDA (Intervention calculus when the DAG is absent) without (**a**) and with (**b**) chronological a priori information for 2 variables *X*_*i*_,*X*_*j*_ measured at 3 time points *t*_1_,*t*_*2*_ and *t*_3_.

This can be done by adding chronological order information among the variables in addition to the conditional independence information as input of the PC-stable algorithm. The result of combining these two types of information can be viewed as a partially directed graph. In the partially directed graph, all edges between variables measured at different times should be directed chronologically, from parents to descendants, and edges between variables measured at the same time remained undirected. Differences between the two initial graphs with 2 variables measured at 3 time points are shown in Fig. 2. A global sketch of the chronologically ordered PC-stable is shown in algorithm 2.

The modified step 1 leads to determine a *partially directed skeleton* at the end of step 1. We will call this extension of PC-algorithm chronologically ordered PC-stable (COPC-stable) when using the order-dependent version or the COPC- algorithm when not. The R code of this version is available on request.

#### Estimation of the causal effect of repeated continuous covariates on a binary outcome

The estimation of causal effects for data with only continuous or only discrete data has been largely discussed [3, 22]. In estimate causal effect of repeated continuous covariates on a binary outcome, the collinearity may address the issue of unstable maximum likelihood estimates. Therefore we used the Firth’s correction to address this problem [23, 24]. Our model is detailed in the Additional file 1.

### Simulations

To compare our algorithm COPC to the PC-stable algorithm, we used simulations. We generated random weighted DAGs with a given number of variables *p* per visit, a given number of visits *n*_*visits*_(corresponding to measurements of these variables) and a single binary outcome. To simulate collinearity between repeated measures, we generated the repeated covariates data from a multivariate distribution that uses an autoregressive model for the correlation between biomarkers:

$$ X\sim N\left(\mu =\left(\begin{array}{c}{\mu}_1\\ {}\vdots \end{array}{\mu}_{n_{visits}}\right),\Sigma =\left(\begin{array}{ccc}{\rho}^0{\sigma}^2& \cdots & {\rho}^{n_{visits}}{\sigma}^2\\ {}\vdots & \ddots & \vdots \\ {}{\rho}^{n_{visits}}{\sigma}^2& \cdots & {\rho}^0{\sigma}^2\end{array}\right)\right), $$

where *ρ* is the correlation between biomarkers. We choose to set *μ* = 0, *σ*^2^ = 1 and vary *ρ* from 0.5 to 0.7. We also tried different number of visits and observations from 3 to 6 and 50 to 1000 respectively. To evaluate the two methods, we compared the capacity of recovering the true CPDAG through the sensibility and the specificity which determine the capacity of detecting the true presence of an arrow and the true absence of an arrow respectively. We also calculated the Structural Hamming distance (SHD) described by Tsamardinos [25] which is a score to evaluate the structural distance from an estimated graph to a true graph. The SHD was calculated as follows: SHD was incremented when there was a wrong connection (i.e. there was an arrow in the estimated CPDAG that was absent in the true CPDAG), and a missed edge (i.e. there was no arrow in the estimated CPDAG that was present in the true CPDAG). The accuracy of the causal effects estimation was explored by calculating the mean squared errors (MSE). The full details of the simulations set-up are available in Additional file 2.

### Application

The method described above was applied to observational data of repeated immunological biomarkers from patients treated with ipilimumab for metastatic melanoma. The objective was to highlight immunologic biomarkers that had a causal effect on early toxicity, premature death and progression.

#### Patients

Patients with metastatic melanoma treated with ipilimumab were prospectively enrolled at the Gustave Roussy Cancer Campus. Ipilimumab was administered intravenously every 3 weeks. Immunological biomarkers were measured at each visit prior each ipilimumab infusion (V_1,_ V_2_, V_3_, and V_4_).

#### Outcomes

Three binary outcomes were investigated: toxicity, premature death and progression. Early toxicity was defined as occurrence of colitis during the first 12 weeks of treatment. Premature death referred to death during the first 12 weeks of treatment. Progression was defined as an increase of at least 20% in tumor size or occurrence of new lesions during the first 6 months of treatment.

#### Immunological biomarkers

Several biological models were used representing different level of immunological expression (Table 1). Model 1 represents adaptive T cells in a global way while model 3 represents subgroup of adaptive T cells. In all three models biomarkers with a potentially known effect were incorporated. For convenience, all biomarkers have been anonymised in the main text of this page but are fully detailed in Additional file 3.

**Table 1:** Biological models representing different level of immunological expression

Model	Common biomarkers (n)	Adaptive T cells immunity biomarkers (n)	Total number of covariates
Model 1	Non-immunologic and innate immunological biomarkers (29)	CD4 and CD8 (8)	37
Model 2	Non-immunologic and innate immunological biomarkers (29)	CD4\CD8 expressing polarization and domiciliation markers (148)	177
Model 3	Non-immunologic and innate immunological biomarkers (29)	Subgroup of CD4\CD8 expressing polarization and domiciliation markers (232)	261

#### Representation

To identify the dependency structure of the data, CPDAGs were estimated using the PC-algorithm. To resume the (conditional) dependencies present in all CPDAGs, Kalisch et al. [14] proposed to aggregate edges in a present in CPDAGs from a resampled dataset rather than showing a single estimate of the CPDAG. Only edges present in 20% of the CPDAGs are drawn and their thickness is proportional to the number of CPDAGs in which the edge was present.

#### Missing data

In our melanoma application, around 15% of missing data were imputed using multivariate imputation by chained equations (MICE) [26]. Missingness graphs [27] are substantives tools that have been developed to study the missingness mechanisms and the recoverability of a missing variable. We applied missingness graphs on our data in order in to identify the missingness mechanisms and the recoverability. In missing at random (MAR) case, the missing values can be recovered without bias; while in the missing not at random (MNAR) case, the missing values could be recovered with some little bias. Full details are provided in the Additional file 4.

## Results

### Simulations

The results of the simulations are presented in Table 2.

Overall, COPC-stable outperformed PC-stable in terms of sensibility, meaning that the percentage of false positive was lower in the CPDAGs estimated with COPC-stable rather than the CPDAGs estimated with PC-stable. In terms of specificity, both algorithms showed excellent results. In scenarios with a greater alpha level regarding other parameters, sensibility rose while specificity decreased. By reducing the number of observations from 1000 to 50 we underestimated slightly the sensitivity and specificity.

**Table 2:** Average sensibility, specificity and SHD according PC-stable and COPC-stable over 500 replicates simulated based on 2 DAGs with different number of visits

n_visits_	n_obs_	alpha	Se PC-stable (sd) %	Se COPC-stable (sd) %	Sp PC-stable (sd) %	Sp COPC-stable (sd) %	SHD PC-stable (sd)	SHD COPC-stable (sd)
4	1000	0.02	58.1(0.6)	63.2 (0.5)	98.7 (0.1)	98.8 (0.1)	333 (9)	279 (7)
		0.2	58.1(0.5)	64.1(0.5)	98.6 (0.1)	98.5 (0.1)	340 (9)	288 (8)
	50	0.02	54.9 (0.5)	57.0 (0.6)	99.2 (0.1)	99.4 (0.1)	338 (8)	299 (9)
		0.2	56.3 (0.6)	59.0 (0.6)	98.9 (0.1)	99.1(0.1)	339 (9)	296 (8)
6	1000	0.02	56.6 (0.4)	60.7 (0.4)	99.0 (0.1)	98.9 (0.1)	504 (10)	466 (8)
		0.2	56.6 (0.4)	61.6 (0.4)	98.9 (0.1)	98.6 (0.1)	521 (9)	491 (10)
	50	0.02	54.1(0.3)	55.6 (0.4)	99.4 (0.1)	99.5 (0.1)	484 (9)	455 (11)
		0.2	55.2 (0.4)	57.5 (0.5)	99.2 (0.1)	99.3 (0.1)	494 (11)	454 (12)

The COPC-stable SHD was lower than the PC-stable in all scenarios, meaning that, as compared with CPDAGs estimated with PC-stable, CPDAGs estimated with COPC-stable had a structure closer to the true CPDAG (see Table 2).

In terms of accuracy, the estimations of causal effects based on CPDAGs estimated with COPC-stable were more accurate than the ones using CPDAGs estimated with PC-stable (see Additional file 5 for results of all scenarios).

### Application

Both IDA and our extension have been applied to our observational data of repeated immunological biomarkers from patients treated with immunotherapy for metastatic melanoma. They have been repeatedly run 300 times on subsamples of size *n* = 30. The tuning parameter α was set to 0.02.

**Fig. 3:**
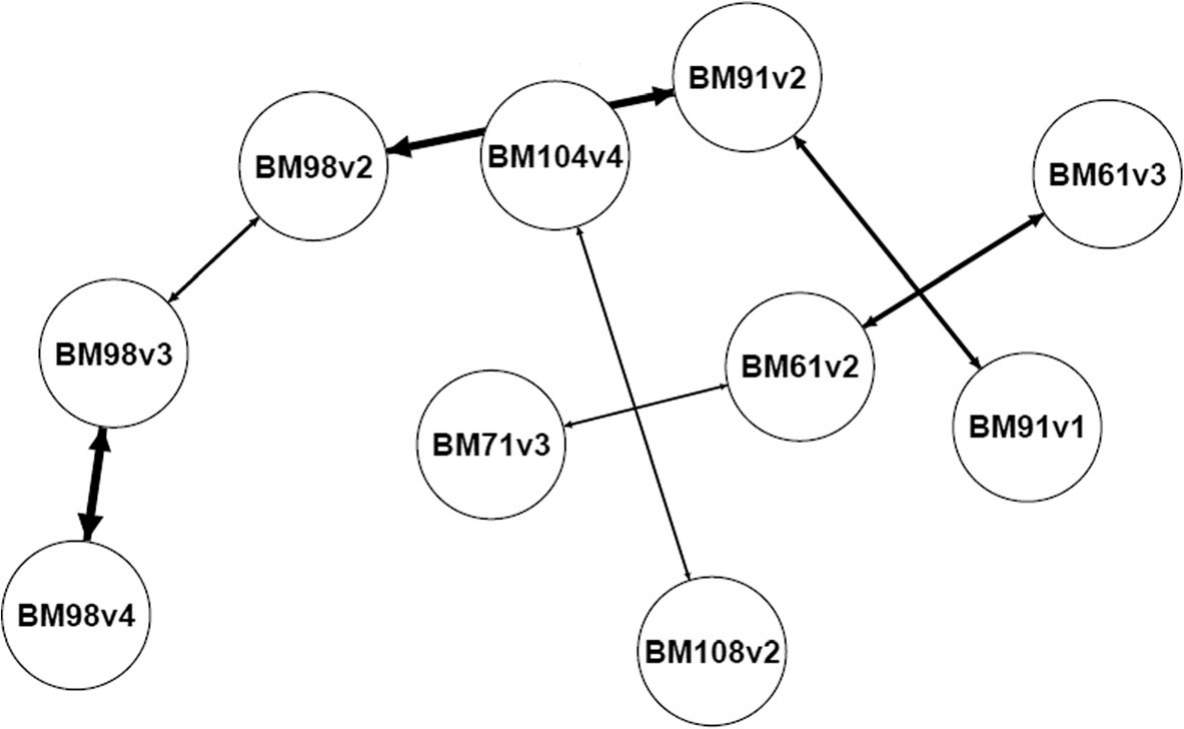
Subset of the summary CPDAGs (Completed partially DAGs) of the model 3 in the metastatic melanoma example using naive PC-stable over 300 runs. Only edges with a frequency > 0.20 are present. The thickness of edges is proportional to their frequency

As expected, CPDAGs obtained using a naive PC-stable from unordered repeated measures led to non-chronological ordered paths in all three models (Fig. 3) as compared with paths identified through COPC.

Table 3 shows the average number of edges according to the version of the PC-algorithm and the model. As compared with PC-stable, the percentage of bidirected edges among all edges using COPC-stable was on average smaller in all three models, 100% vs 28% for model 1, 98% vs 40% for model 2 and 97% vs 52% for model 3. Moreover, Table 3 shows the number of wrongly defined edges into the final CPDAG. For instance, in model 1, when using a naive approach of the PC-stable, the resulting CPDAG had on average 14 bidirected edges that were between two variables measured at different times. When looking at Table 4, the number of values in each multiset *Θ*_*i*_ also called *ambiguity* (*â*) of the multiset [1] was smaller when using COPC-stable rather than PC-stable for a same value of alpha (*α* = 0.02). The maximum *ambiguity* reached in our application was 3.

**Table 3:** Average number of edges (standard deviation) in the CPDAG according to the version of the PC-algorithm and the model over 300 runs with alpha = 0.02

	Directed edges (sd)	Bidirected edges (sd)	Total (sd)	Non-chronologically ordered edges (sd)
Naïve PC-stable (model 1)	0 (0.1)	19 (0.1)	19 (0.1)	14 (0.1)
COPC-stable (model 1)	18 (0.1)	7 (0.1)	25 (0.1)	0 (0)
Naïve PC-stable (model 2)	1 (0.1)	94 (0.1)	95 (0.1)	53 (0.1)
COPC-stable (model 2)	78 (0.1)	52 (0.1)	130 (0.1)	0 (0)
Naïve PC-stable (model 3)	5 (0.2)	155 (0.1)	160 (0.2)	64 (0.1)
COPC-stable (model 3)	92 (0.2)	100 (0.1)	192 (0.2)	0 (0)

**Table 4:** Probability of having a certain ambiguity *â* for biomarkers with an alpha level at 0.02 according to the version of the PC-algorithm (PC-Stable/ COPC-stable) over 300 with alpha = 0.02

	Model 1		Model 2		Model 3	
Ambiguity	PC-stable	COPC-stable	PC-stable	COPC-stable	PC-stable	COPC-stable
â=1	0.243	0.676	0.153	0.599	0.061	0.437
â=2	0.568	0.297	0.655	0.356	0.693	0.494
â =3	0.189	0.027	0.192	0.045	0.245	0.069

#### Estimating time-dependent causal effects in the melanoma example

**Fig. 4:**
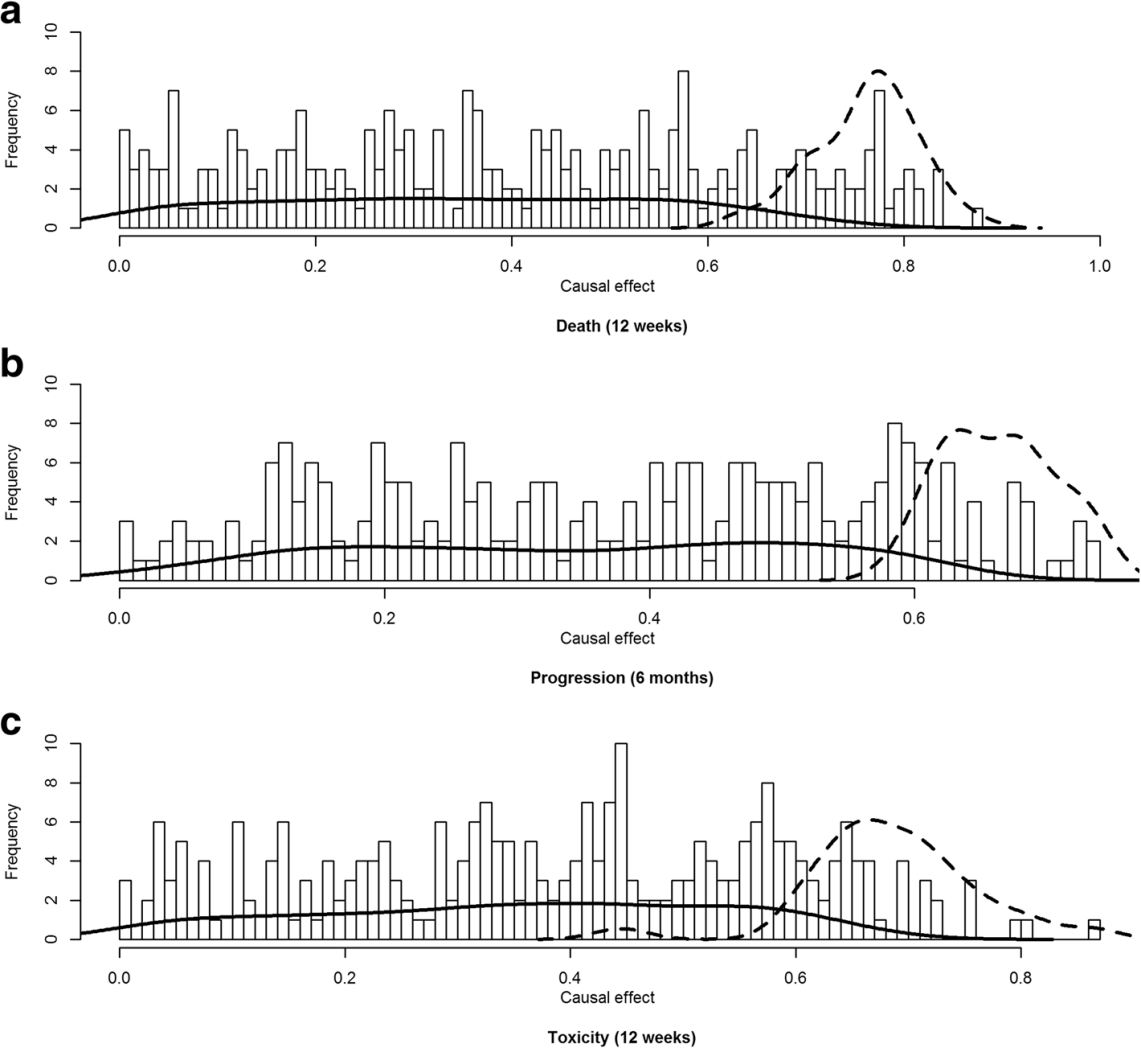
Histogram of the causal effect for the biomarkers on death (**a**), progression (**b**) and toxicity (**c**) based on model 2 over 300 runs. Solid and dashed lines represent the kernel density of biomarkers with a PCER > 0.5% and PCER ≤0.5% respectively

**Fig. 5:**
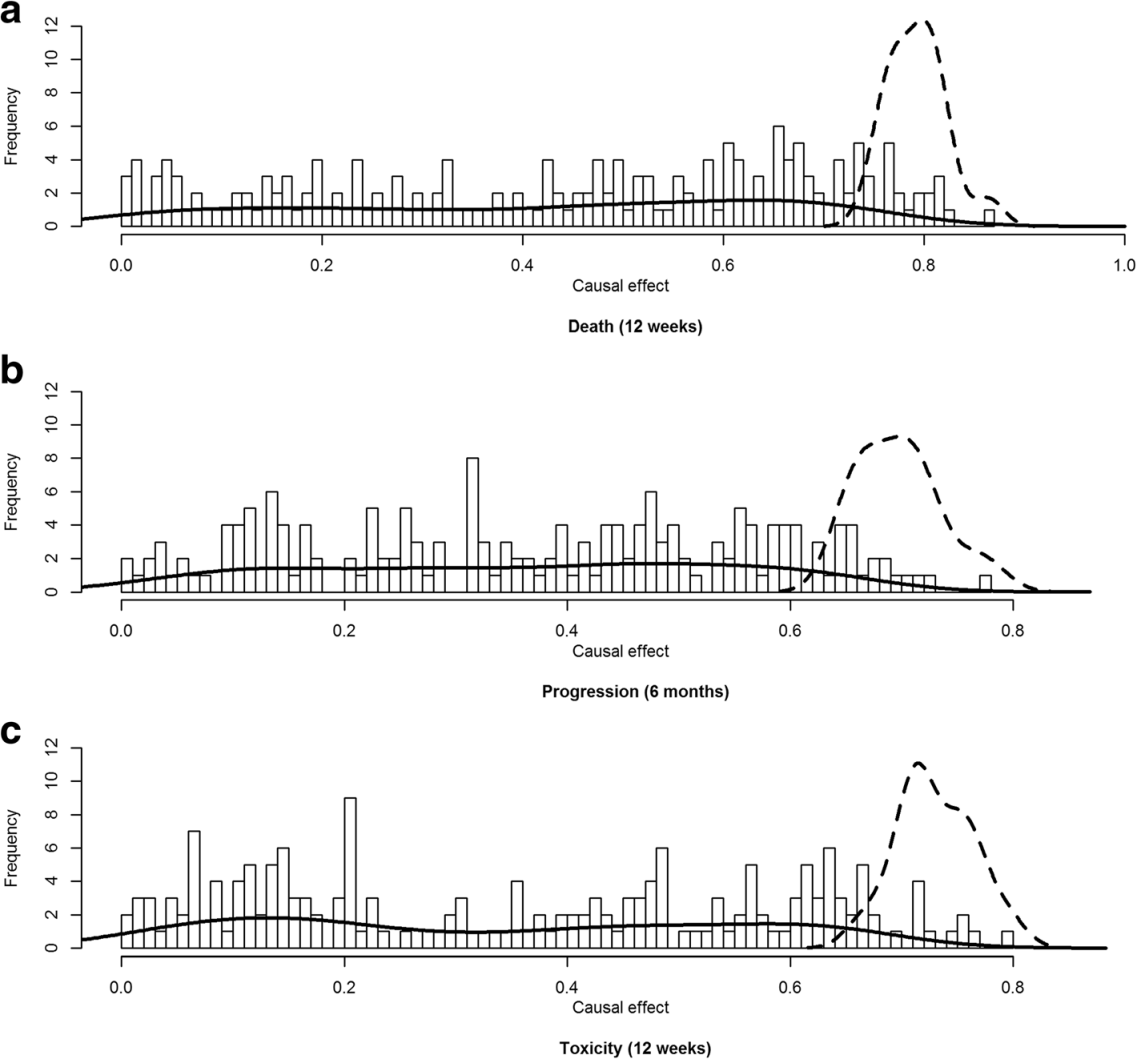
Histogram of the causal effect for the biomarkers on death (**a**), progression (**b**) and toxicity (**c**) based on model 3 over 300 runs. Solid and dashed lines represent the kernel density of biomarkers with a PCER > 0.5% and PCER ≤0.5% respectively

After estimating the CPDAG using COPC-stable, causal effects were estimated using Pearl’s *do*-calculus. To determine which biomarker had a robust causal effect, we intended to select biomarkers with PCER threshold ≤0.5%. In model 1, there were no biomarkers with a PCER < 0.005. Figures 4 and 5 show histograms of causal effects on our three outcomes death, progression and toxicity based on model 2 and 3. The causal effects seem almost uniformly distributed between 0 and 1 in our example for models 2 and 3. However, immunological biomarkers with a PCER under 0.5% had a causal effect concentrated between 0.6 and 0.8 for models 2 and 3 for all outcomes. On the other hand, causal effects sizes of immunological biomarker with PCER > 0.5% were spread in a wide range from 0 to 1.

Tables 5 shows the top effect biomarkers among those selected for models 2 (see Additional file 6 for the list of all selected immunological biomarkers).

We see that some of the biomarkers are present in all top 10 but differ with the time of measurement. We see that BM30 is present in the top 10 for toxicity at visit 1, in the top 10 for progression at visit 3 and in the top ten for death at visit 4. Other biomarkers are present in 2 of the top 3 but differ with the visit such as BM26, BM45, BM39 and BM9.

**Table 5:** Top 10 of immunological biomarkers with a PCER < 0.5% in model 2. The number following “v” stands for the visit number. Superscript indicate biomarkers in common. See Additional file 3 for the complete description of the biomarkers

	Death (12 weeks)			Progression (6 months)			Toxicity 12 weeks		
Rank	Biomarker	Median effect	PCER	Biomarker	Median effect	PCER	Biomarker	Median effect	PCER
1	BM16v2^a^	0.81	0.0035	BM8v1^d^	0.77	0.0031	BM7v4	0.79	0.0028
2	BM5v1	0.81	0.0035	BM44v4	0.72	0.0036	BM8v4^d^	0.76	0.0034
3	BM42v3	0.80	0.0037	BM26v2	0.71	0.0041	BM16v3^a^	0.75	0.0036
4	BM48v1	0.86	0.0037	BM30v3^b^	0.71	0.0041	BM26v4	0.75	0.0036
5	BM42v2	0.80	0.0038	BM44v3	0.68	0.0042	BM7v3	0.76	0.0037
6	BM14v4	0.79	0.0039	BM45v1^e^	0.70	0.0047	BM9v4^c^	0.72	0.0039
7	BM30v4^b^	0.80	0.0039	BM39v4^f^	0.66	0.0049	BM39v3^f^	0.72	0.0039
8	BM11v4	0.83	0.0040	BM40v4	0.66	0.0049	BM32v3	0.71	0.0042
9	BM11v1	0.76	0.0042	BM14v2	0.66	0.0050	BM30v1^b^	0.67	0.0045
10	BM9v4^c^	0.81	0.0043	–	–	–	BM45v1^e^	0.71	0.0046

## Discussion

We extended in this paper the IDA method to repeated measures by introducing a chronologically ordered (CO) version of the so called PC-algorithm. Our proposed algorithm COPC-algorithm takes a priori chronological information, such as repeated measure, into account in the input graph. We then applied PC-stable and our new method COPC-stable to simulated data sets and observational data of repeated immunological biomarkers from patients treated repeatedly with immunotherapy for metastatic melanoma. When comparing CPDAGs obtained with PC-stable and those with COPC-stable, the simulation study showed that PC-stable had a lower sensitivity than the COPC-stable leading to a better learning of the true structure. On the application, CPDAGs based on PC-stable had indeed non-chronological ordered paths while those based on COPC-stable could not have any. CPDAGs obtained with COPC-stable had on average more total and directed edges than those obtained with PC-stable but less bidirected edges. The lower the number of directed edges, the lower the number of possible ways to direct edges, hence the lower the number of DAGs in the *Markov equivalence class*. Moreover Table 4 showed that when using COPC-stable, the proportion of values obtained in the multiset *Θ*_*i*_ was on average lower when using PC-stable. Smaller the *Markov equivalence class*, higher the power of the study to identify causal effects.

In the COPC-stable, the number of tested conditional dependencies is considerably smaller than with PC-stable. Since it takes chronological order information into account, the COPC-algorithm does not test dependencies of two variables conditioning on a variable measured at a time after those two variables. In contrary, the original PC-algorithm tests non-realistic conditional dependences and thus raises the number of global tests. Testing those non-realistic conditional dependences could lead to identifying false positive causal effects.

Finding the true causal DAG has always been the principle interest of causal inference studies, knowing the true causal DAG allows the estimating of the true causal effect. However, in high-dimensional setting, the true causal DAG is generally unknown and it is difficult to check whether or not all possible confounders are measured. Therefore IDA was developed to estimate lower bounds of the causal effects of *X*_*i*_ on *Y* and determine the importance of these effects. This is a different approach where instead of searching one true causal effect, a range of causal effects are estimated in each DAG from a *Markov equivalence class*. Consequently, when an effect of large numbers of markers is identified, those which have causal effects could be selected by different approaches. In fact, we could either keep a small range of biomarkers that are in the top effects as in [21] or a larger range of those with a limited but slightly higher probability of being false positive. In the high-dimensional setting, the first approach will keep biomarkers with the strongest causal effect but not necessarily all biomarkers with a small causal effect. The second approach assures to select a larger list of biomarkers that have a robust causal effect and will suggest to clinicians which immunological biomarkers they should investigate deeper in a follow-up study. Also, controlling for type 1 error can be done by different methods. We chose in our application the PCER because it is less restrictive compared to methods such as FDR (False discovery rate) or FWER (Family-wise error rate).

The choice of the selecting approach depends on the objective: selecting a small list of biomarker that have the highest effect on the outcome or identifying all the biomarkers that have an effect regardless of the size effect. For instance, if only the measure of a marker at visit 2 belongs to the top causal effects, should we only consider the marker at visit 2 or should the marker be measured at all visits? Usually, in a causal DAG, all true causal effects have to be reported, not only the strongest. Nevertheless, the interpretation of the top causal biomarker is challenging. Having a biomarker at a certain visit with a PCER below the selected threshold does not mean that the biomarker has a causal effect only at this visit but rather its maximum and more robust effect at this visit.

One of the main assumptions made in this study is that the true DAG is not dynamic like other extensions of the PC-algorithm on time-series data [28, 29]. So we did not constrain the arrows to be the same within each visit. In fact, the context of biological biomarkers can be much more complex than a simple repetition of a pattern. Originally, the IDA made the assumption that all variables including the outcome were Gaussian, then it has been extended in a case where all variables (including outcome) are discrete [14]. In this study we made the assumption that all covariates *X* = {*X*_1_, …, *X*_*p*_} are Gaussian and that the outcome is binary because it is a situation that is quite common in oncology. Also, the covariates need to be measured at uniform set of time points (i.e. balanced data).

Our work aimed to find causal effects among repeated immunological biomarkers on death and toxicity of patients treated with immunotherapy for metastatic melanoma. Based on our observational data, using the IDA with our new version of the PC-algorithm, the COPC-algorithm, we found a consistent list of immunological biomarkers with causal effects. But one should be attentive not to overinterpret these results. It is in fact impossible to accurately check whether or not our assumptions hold; having no unmeasured confounders is a strong assumption but may be reasonable in our application.

Further work will investigate the addition of expert knowledge as input of the COPC-algorithm based on high-dimensional graphs. Also we will explore extensions that can deal with longitudinal and time to event outcomes.

## Conclusions

In this paper, we presented an extension of the PC-algorithm called COPC-algorithm. It provides CPDAGs that keep the chronological structure present in the data and allow us to estimate reliable lower bounds of the causal effect of repeated covariates or biomarkers. In the immunotherapy example, immunological biomarkers on early toxicity, premature death and progression were identified and will be further investigated by clinicians.

## Additional files

Additional file 1: Estimation of causal effect. It details the formulas used for the calculation of causal effects. (DOCX 26 kb)

Additional file 2: Simulation setting. It details the process of the generation of the data for the simulation study. (PDF 113 kb)

Additional file 3: Description of biomarkers. It describes the names of the anonymised biomarkers. (DOCX 30 kb)

Additional file 4: Missingness graphs. It details the process used to recover missing data. (DOCX 212 kb)

Additional file 5: Estimation of the Mean squared error. It provides supplementary tables of the simulation study results. (DOCX 27 kb)

Additional file 6: Estimation of biomarkers’ median effect. It provides supplementary tables of the application results. (DOCX 31 kb)

## Abbreviations

COPC-algorithm: Chronologically ordered PC-algorithm
COPC-stable: Chronologically ordered PC-stable
CPDAG: Completed partially Directed Acyclic Graph
CStaR: Causal Stability Ranking
DAG: Directed Acyclic Graph
FDR: False discovery rate
FWER: Family-wise error rate
IDA: Intervention calculus when the DAG is Absent
MSM: Marginal Structural Model
PC-algorithm: Peter Clarks -algorithm
PCER: Per comparison error rate
SHD: Structural Hamming Distance
