# Supplementary material for: Estimating causal effects of time-dependent exposures on a binary endpoint in a high-dimensional setting
Source: BMC Med Res Methodol. 2018 Jul 3;18:67. doi: 10.1186/s12874-018-0527-5 (PMC6029422; doi:10.1186/s12874-018-0527-5)
Supplement: Supplementary file 1 — Estimation of causal effect. It details the formulas used for the calculation of causal effects. (DOCX 26 kb) [file 12874_2018_527_MOESM1_ESM.docx]

**Estimation of the causal effect of a repeated biomarker on a binary outcome**

Causal effects of $X_{i}$ on the outcome $Y$ can be quantified by measuring the difference of any function of the distribution of “counterfactuals outcome” such as mean, median or ratios (see technical point 1.1 of [1]). Let *G* be a directed acyclic graph with V the set of nodes described by $p + 1$ variables$X_{1},\ldots,X_{p},Y$.

Pearl [2] showed that the distribution generated from a DAG is called Markovian and can be factorized as

$$\left( 1 \right) P\left( x_{1},\ldots,x_{p},Y=x_{p}+1 \right)=\prod_{i=1}^{p+1} P\left( x_{i} | pa\left( x_{i} \right) \right).$$

Intervention on a variable using Pearl’s do operator assigns a value to the variable over the population; and the distribution generated on the variables set can be expressed in the *truncated factorization formula*:

$$\left( 2 \right) P\left( x_{1},\ldots,x_{p},Y=x_{p}+1|do(X_{j}={x'}_{j} \right)=\left\{ \begin{aligned} \prod_{i=1,i\neq j}^{p+1} P\left( x_{i} | pa\left( x_{i} \right) \right) ifx_{j}={x'}_{j} \\ 0 ifx_{j}\neq{x^{'}}_{j}. \end{aligned} \right.$$

Equation (2) reflects the removal in (3) of $P\left( x_{i} | pa\left( x_{i} \right) \right)$ since $pa\left( x_{i} \right)$ has no effect on$x_{i}$. Graphically, removing $P\left( x_{i} | pa\left( x_{i} \right) \right)$ is equivalent to removing the arrows from $pa\left( x_{i} \right)$ to$x_{i}$.

Based on (2), Pearl’s has shown that the effect of the intervention $do(X_{i}={x^{'}}_{i})$ on Y is given by

$$\left( 3 \right) P(Y|do\left( X_{j}={x^{'}}_{i} \right)=\sum_{pa\left( x_{i} \right)} P(Y|do\left( X_{i}={x^{'}}_{i} \right),pa\left( x_{i} \right) P(pa\left( x_{i} \right)).$$

Equation (3) means conditioning $P(Y|do\left( X_{j}={x^{'}}_{i} \right)$ on the parents of $X_{i}$ and then averaging the results weighted by the probability of $pa\left( x_{i} \right)$.

For a continuous outcome, $P(Y|do\left( X_{j}={x^{'}}_{i} \right)=E\left( Y | do\left( X_{i}=x \right) \right)$, which denotes the mean of $Y$ when $X_{i}$ is uniformly assigned to $x$ over the population. So we can defined the average causal effect for a continuous outcome *Y* by

$$\left( 4 \right) E\left( Y | do\left( X_{i}=x \right) \right)-E\left( Y | do\left( X_{i}={x'}_{i} \right) \right),$$

where $E(Y|do\left( X_{i}=x_{i} \right))$ and $E\left( Y | do\left( X_{i}={x'}_{i} \right) \right)$ denote the mean of $Y$ when $X_{i}$ is uniformly assigned to$x_{i}$ or ${x'}_{i}$ over the population via Pearl’s *do* operator. In the case of continuous Gaussian variables, the causal effect of $X_{i}$ on *Y* is the regression coefficient $\beta_{1}$ of $X_{i}$ in the linear regression of *Y* on $X_{i}$ and *pa*(*X_i_,G*) [1, 2]:

$$\left( 5 \right) E\left( Y | X_{i},pa\left( X_{i},G \right) \right)= \beta_{0}+\beta_{1}X_{i}+ \beta_{{pa}_{i}}{pa}_{i}.$$

However, in the case where covariates $X_{1},\ldots,X_{p}$are Gaussian and the outcome Y is binary, the linearity stated in (5) does not hold. This is why we modelled the relation using a logit link that allows keeping the linearity and calculating the causal effect for a binary outcome as

$$\left( 4 \right) logit P\left( Y=1 | X_{i},pa\left( X_{i},G \right) \right)=\beta_{0}+\beta_{1}X_{i}+ \beta_{{pa}_{i}}{pa}_{i}.$$

This method of modelling is widely used in [1] (see technical point 11.1).

However, the “separation” phenomenon [3] could occur in small datasets in logistic regression such as in our melanoma example: subjects having $y=1$ and subjects having $y=0$ can be separated by a single or a combination of covariates, the likelihood converges while at least one parameter estimate diverges to$\pm\infty$, leading to infinite odds ratio estimates. To overcome this situation, Firth proposed to reduce the bias of maximum likelihood estimates [4]. Several studies have shown that this method provides unbiased estimates [5, 6]. In our small observational dataset setting, the causal effects on dichotomous outcome will be estimated through logistic regression with Firth correction.

1. Hernan MA, Robins JM: *Causal Inference*. Boca Raton: Chapman & Hall/CRC; 2016.

2. Pearl J: *Causality: Models, Reasoning, and Inference*. 2nd ed. Cambridge university press; 2009.

3. Albert A, Anderson JA: **On the existence of maximum likelihood estimates in logistic regression models**. *Biometrika* 1984, **71**:1–10.

4. Firth D: **Bias Reduction of Maximum Likelihood Estimates**. *Biometrika* 1993, **80**:27–38.

5. Heinze G, Schemper M: **A solution to the problem of separation in logistic regression**. *Stat Med* 2002, **21**:2409–2419.

6. Heinze G: **A comparative investigation of methods for logistic regression with separated or nearly separated data**. *Stat Med* 2006, **25**:4216–4226.
