# Supplementary material for: Estimating causal effects of time-dependent exposures on a binary endpoint in a high-dimensional setting
Source: BMC Med Res Methodol. 2018 Jul 3;18:67. doi: 10.1186/s12874-018-0527-5 (PMC6029422; doi:10.1186/s12874-018-0527-5)
Supplement: Supplementary file 2 — Simulation setting. It details the process of the generation of the data for the simulation study. (PDF 113 kb) [file 12874_2018_527_MOESM2_ESM.pdf]

Simulation set-up

In this additional file, we explain how we run our simulations and what scenarios we tested. In our scenarios, we fixed the number of variables, of maximum parent per node and the correlation between biomarkers. We tested different value for the number of observations, the number of measurement (visits) and for the independence test cut-off alpha leading to a total of 8 scenarios.

Scenarios

We generated data faithful to a by using the following parametrisations:

- $p = 20$
- $n_{visits} \in \{4, 6\}$
- $maxP = 3$
- $n \in \{50, 1000\}$
- $\alpha \in \{0.02, 0.2\}$
- $\rho_{min} = 0.5$, $\rho_{max} = 0.7$
- $\sigma = 1$

For each scenario we generated a DAG with $p * n_{vis} + Y$ variables and then sampled 500 different datasets per DAG. We calculated the average SHD, sensibility and specificity for estimated CPDAGs with both CP-stable and COPC-stable based on the 500 datasets.

Generate a DAG with repeated measures of covariates

To generate a DAG that has repeated measures and an expected number of parents per node $maxP$, we used the following approach: first, we generated a $(p \times n_{visits} + Y) \times (p \times n_{visits} + Y)$ weighted matrix wM, where p is the number of biomarkers, n_{visits} the number of visits (measurements) and Y the outcome; with independent realizations of $Uniform(0.5, 1)$ in the upper triangle of the matrix and zeroes in the remaining entries. At the end, if $wM_{i,j} > 0$ then, it meant there was an arrow from X_i to X_j ($X_i \rightarrow X_j$) with a weight of $wM_{i,j}$. The maximum parent for each node was limited by $maxP$. Then, based on the true DAG obtained, we generated i.i.d samples using algorithm 1.

Algorithm 1: Generation of data based on a DAG with repeated measures of covariates with a single outcome

Input: number of observations (n), weighted matrix (wM), sigma (σ), ρ_{min}, ρ_{max}, number of biomarkers (p), number of measurements (n_{visits})

for Subject $i \leftarrow 1$ **to** n **do**

  for Number of Biomarkers $j \leftarrow 1$ **to** p **do**

    Let V_j be the set of measurements for the biomarker j

    Let the vector $\mu_{V_j} = E(V_1) = E(V_2) = E(V_{n_{visits}}) = 0$

    $\rho_j \sim Uniform(\rho_{min}, \rho_{max})$

$$\Sigma_j = \begin{pmatrix} \sigma^2 & \rho^1\sigma^2 & \rho^2\sigma^2 & \dots & \rho^{n_{visits}}\sigma^2 \\ \rho^1\sigma^2 & \sigma^2 & \rho^1\sigma^2 & \dots & \rho^{n_{visits}-1}\sigma^2 \\ \rho^2\sigma^2 & \rho^1\sigma^2 & \sigma^2 & \dots & \rho^{n_{visits}-2}\sigma^2 \\ \vdots & \vdots & \vdots & \ddots & \vdots \\ \rho^{n_{visits}}\sigma^2 & \rho^{n_{visits}-1}\sigma^2 & \rho^{n_{visits}-2}\sigma^2 & \dots & \sigma^2 \end{pmatrix} \epsilon_{i,V_j} \sim N_{n_{visits}}(\mu_j, \Sigma_j)$$

    $\epsilon_{i,Outcome} \sim N(0,1)$

  end

end

$p_{tot} = p \times n_{visits} + 1$ (outcome)

for Subject $i \leftarrow 1$ **to** n **do**

  for Variable $v \leftarrow 1$ **to** p_{tot} **do**

    if $Variable_v$ has no parent **then**

      $Variable_{i,v} = \epsilon_{Variable_{i,v}}$

    end

    else if $Variable_v$ has at least 1 parent **then**

      $Variable_{i,v} = \sum_{j=1}^{n_{parents}} (wM_{j,v} \times PA_j(Variable_{i,v})) + \epsilon_{Variable_{i,v}}$

      if $v = p$ **then**

        $Variable_{i,p} \sim Bernoulli(logit^{-1}(Variable_{i,p_{tot}}))$

      end

    end

  end

end

Output: Simulated database
