# Supplementary material for: Estimating causal effects of time-dependent exposures on a binary endpoint in a high-dimensional setting
Source: BMC Med Res Methodol. 2018 Jul 3;18:67. doi: 10.1186/s12874-018-0527-5 (PMC6029422; doi:10.1186/s12874-018-0527-5)
Supplement: Supplementary file 3 — Description of biomarkers. It describes the names of the anonymised biomarkers. (DOCX 30 kb) [file 12874_2018_527_MOESM3_ESM.docx]

**Description of all anonymised biomarkers of the application**

**Table 1:** Description of the anonymised biomarkers for model 1. The number following “v” stands for the visit number.

| Labels | Description |
| --- | --- |
| BM 1v1 | Interleukin 6 |
| BM 2 v1 | Interleukin 8 |
| BM 3 v1 | Monocyte chemo attractant protein 1 |
| BM 4 v1 | Interferon gamma-induced protein 10 |
| BM 5 v1 | Tumour Necrosis Factor α |
| BM 6 v1-v4 | C reactive protein |
| BM 7 v1-v4 | Lactate Dehydrogenase |
| BM 8 v1-v4 | Eosinophils |
| BM 9 v1-v4 | Neutrophils |
| BM 10 v1-v4 | Soluble CD25 |
| BM 11 v1-v4 | Soluble CTLA-4 |
| BM 12 v1-v4  BM 13 v1-v4 | CD4^+^ T cells  CD8^+^ T cells |

**Table 2:** Description of the anonymised biomarkers for model 3. The number following “v” stands for the visit number.

| Labels | Description | |
| --- | --- | --- |
| BM 1v1 | Interleukin 6 | |
| BM 2 v1 | Interleukin 8 | |
| BM 3 v1 | Monocyte chemo attractant protein 1 | |
| BM 4 v1 | Interferon gamma-induced protein 10 | |
| BM 5 v1 | Tumour Necrosis Factor α | |
| BM 6 v1-v4 | C reactive protein | |
| BM 7 v1-v4 | Lactate Dehydrogenase | |
| BM 8 v1-v4 | Eosinophils | |
| BM 9 v1-v4 | Neutrophils | |
| BM 10 v1-v4 | Soluble CD25 | |
| BM 11 v1-v4 | Soluble CTLA-4 | |
| BM 14 v1-v4 | % of Foxp3^+^CD25^+^ among CD4^+^ T cells | |
| BM 15 v1-v4 | % of conventional CD4^+^ T cells (Tconv) (FoxP3^-^) | |
| BM 16 v1-v4 | % of CD49d^+^β7^+^ among CD4^+^ T cells | |
| BM 17 v1-v4 | % of CD49d^+^β7^+^ among CD4^+^ T cells | |
| BM 18 v1-v4 | % of β7^+^CD103^+^ among CD4^+^ T cells | |
| BM 19 v1-v4 | % of C-C chemokine receptor 6^+^(CCR6^+^) among CD4^+^ T cells | |
| BM 20 v1-v4 | % of Cutaneous Lymphocyte-associated antigen^+^ (CLA^+^) C-C chemokine receptor 10^+^ (CCR10^+^) among CD4^+^ T cells | |
| BM 21 v1-v4 | % of CLA^+^ among CD4^+^ T cells | |
| BM 22 v1-v4 | % of central memory among CD4^+^ T cells | |
| BM 23 v1-v4 | % of C-X-C chemokine receptor 3^+^(CXCR3^+^) CCR6 ^+^ among CD4^+^ T cells | |
| BM 24 v1-v4 | % of CXCR3^+^ chemokine receptor+ among CD4^+^ T cells | |
| BM 25 v1-v4 | % of C-X-C chemokine receptor 5^+^ (CXCR5^+^) chemokine receptor^+^ among CD4^+^ T cells | |
| BM 26 v1-v4 | % of effector memory among CD4^+^ T cells | |
| BM 27 v1-v4 | % of effector among CD4^+^ T cells | |
| BM 28 v1-v4 | % of memory among CD4^+^ T cells | |
| BM 29 v1-v4 | % of naïve among CD4^+^ T cells | |
| BM 30 v1-v4 | % of CD49d^+^ CD103^+^ among CD8^+^ T cells | |
| BM 31 v1-v4 | % of CD49d^+^ β7^+^ among CD8^+^ T cells | |
| BM 32 v1-v4 | % of β7^+^CD103^+^ among CD8^+^ T cells | |
| BM 33 v1-v4 | % of CCR10^+^ among CD8^+^ T cells | |
| BM 34 v1-v4 | % of CCR6^+^ among CD8^+^ T cells | |
| BM 35 v1-v4 | % of CLA^+^ CCR10^+^ among CD8^+^ T cells | |
| BM 36 v1-v4 | % of CLA^+^ among CD8^+^ T cells | |
| BM 37 v1-v4 | % of central memory among CD8 T cells | |
| BM 38 v1-v4 | % of CXCR3^+^ CCR6^+^ among CD8^+^ T cells | |
| BM 39 v1-v4 | % of CXCR3^+^ among CD8^+^ T cells | |
| BM 40 v1-v4 | % of CXCR5^+^ among CD8^+^ T cells | |
| BM 41 v1-v4 | % of effector memory among CD8^+^ T cells | |
| BM 42 v1-v4 | % of effector among CD8^+^ T cells | |
| BM 43 v1-v4 | % of memory among CD8^+^ T cells | |
| BM 44 v1-v4 | % of naïve among CD8^+^ T cells | |
| BM 45 v1-v4 | % of Inducible CoStimulator (ICOS^+^) among CD8^+^ T cells | |
| BM 46 v1-v4 | % of Inducible CoStimulator (ICOS^+^) among CD4^+^ T cells | |
| BM 47 v1-v4 | % of Inducible CoStimulator Ligand (ICOSL^+^) among CD4^+^ T cells | |
| BM 48 v1-v4 | % of High Inducible CoStimulator Ligand (ICOSL^+^) among CD4^+^ T cells |  |
| BM 49 v1-v4 | % of Inducible CoStimulator Ligand (ICOSL^+^) among CD8^+^ T cells | |
| BM 50 v1-v4 | % of FoxP3^+^ among CD4^+^ T cells | |

**Table 3:** Description of the anonymised biomarkers for model 3. The number following “v” stands for the visit number.

| Labels | Description |
| --- | --- |
| BM 1v1 | Interleukin 6 |
| BM 2 v1 | Interleukin 8 |
| BM 3 v1 | Monocyte chemo attractant protein 1 |
| BM 4 v1 | Interferon gamma-induced protein 10 |
| BM 5 v1 | Tumour Necrosis Factor α |
| BM 6 v1-v4 | C reactive protein |
| BM 7 v1-v4 | Lactate Dehydrogenase |
| BM 8 v1-v4 | Eosinophils |
| BM 9 v1-v4 | Neutrophils |
| BM 10 v1-v4 | Soluble CD25 |
| BM 11 v1-v4 | Soluble CTLA-4 |
| BM 51 v1-v4 | % of CD49d^+^CD103^+^ among central memory CD4^+^ T cells |
| BM 52 v1-v4 | % of CD49d^+^β7^+^ among central memory CD4^+^ T cells |
| BM 53 v1-v4 | % of β7^+^CD103^+^ among central memory CD4^+^ T cells |
| BM 54 v1-v4 | % of CD49d^+^CD103^+^ among effector memory CD4^+^ T cells |
| BM 55 v1-v4 | % of CD49d^+^β7^+^ among effector memory CD4^+^ T cells |
| BM 56 v1-v4 | % of β7^+^CD103^+^ among effector memory CD4^+^ T cells |
| BM 57 v1-v4 | % of CD49d^+^CD103^+^ among effector CD4^+^ T cells |
| BM 58 v1-v4 | % of CD49d^+^β7^+^ among effector CD4^+^ T cells |
| BM 59 v1-v4 | % of β7^+^CD103^+^ among effector CD4^+^ T cells |
| BM 60 v1-v4 | % of CCR10^+^ among effector CD4^+^ T cells |
| BM 61 v1-v4 | % of CCR 6^+^ among effector CD4^+^ T cells |
| BM 62 v1-v4 | % of CLA^+^CCR10 ^+^ among effector CD4^+^ T cells |
| BM 63 v1-v4 | % of CLA^+^ among effector CD4^+^ T cells |
| BM 64 v1-v4 | % of CXCR3^+^ CCR6^+^ among effector CD4^+^ T cells |
| BM 65 v1-v4 | % of CXCR3^+^ among effector CD4^+^ T cells |
| BM 66 v1-v4 | % of CXCR5^+^ among effector CD4^+^ T cells |
| BM 67 v1-v4 | % of CD49+b7+ among memory CD4^+^ T cells |
| BM 68 v1-v4 | % of β7^+^CD103^+^ among memory CD4^+^ T cells |
| BM 69 v1-v4 | % of CCR10^+^ among memory CD4^+^ T cells |
| BM 70 v1-v4 | % of CCR6^+^ among memory CD4^+^ T cells |
| BM 71 v1-v4 | % of CLA^+^ CCR10^+^ among memory CD4^+^ T cells |
| BM 72 v1-v4 | % of CLA^+^ among memory CD4^+^ T cells |
| BM 73 v1-v4 | % of CXCR3^+^ CCR6^+^ among memory CD4^+^ T cells |
| BM 74 v1-v4 | % of CXCR3^+^ among memory CD4^+^ T cells |
| BM 75 v1-v4 | % of CXCR5^+^ among memory CD4^+^ T cells |
| BM 76 v1-v4 | % of CD49d^+^CD103^+^ among naïve CD4^+^ T cells |
| BM 77 v1-v4 | % of CD49d^+^β7^+^ among naïve CD4^+^ T cells |
| BM 78 v1-v4 | % of β7^+^CD103^+^ among naïve CD4^+^ T cells |
| BM 79 v1-v4 | % of CD49d^+^CD103^+^ among central memory CD8^+^ T cells |
| BM 80 v1-v4 | % of CD49d^+^β7^+^ among central memory CD8^+^ T cells |
| BM 81 v1-v4 | % of β7^+^CD103^+^ among central memory CD8^+^ T cells |
| BM 82 v1-v4 | % of CD49d^+^CD103^+^ among effector memory CD8^+^ T cells |
| BM 83 v1-v4 | % of CD49d^+^β7^+^ among effector memory CD8^+^ T cells |
| BM 84 v1-v4 | % of β7^+^CD103^+^ among effector memory CD8^+^ T cells |
| BM 85 v1-v4 | % of CD49d^+^CD103^+^ among effector CD8^+^ T cells |
| BM 86 v1-v4 | % of CD49d^+^β7^+^ among effector CD8^+^ T cells |
| BM 87 v1-v4 | % of β7^+^CD103^+^ among effector CD8^+^ T cells |
| BM 88 v1-v4 | % of CCR10^+^ among effector CD8^+^ T cells |
| BM 89 v1-v4 | % of CCR6^+^among effector CD8^+^ T cells |
| BM 90 v1-v4 | % of CLA^+^ CCR10^+^ among effector CD8^+^ T cells |
| BM 91 v1-v4 | % of CLA^+^ among effector CD8^+^ T cells |
| BM 92 v1-v4 | % of CXCR3^+^ CCR6^+^  among effector CD8^+^ T cells |
| BM 93 v1-v4 | % of CXCR3^+^ among effector CD8^+^ T cells |
| BM 94 v1-v4 | % of CXCR5^+^among effector CD8^+^ T cells |
| BM 95 v1-v4 | % of CCR10^+^ among memory CD8^+^ T cells |
| BM 96 v1-v4 | % of CCR6^+^ among memory CD8^+^ T cells |
| BM 97 v1-v4 | % of CLA^+^ CCR10^+^ among memory CD8^+^ T cells |
| BM 98 v1-v4 | % of CLA^+^ among memory CD8^+^ T cells |
| BM 99 v1-v4 | % of CXCR3^+^ CCR6^+^  among memory CD8^+^ T cells |
| BM 100 v1-v4 | % of CXCR3^+^ among memory CD8^+^ T cells |
| BM 101 v1-v4 | % of CXCR5^+^ among memory CD8^+^ T cells |
| BM 102 v1-v4 | % of CD49d^+^CD103^+^ among naïve CD8^+^ T cells |
| BM 103 v1-v4 | % of CD49d^+^β7^+^ among naïve CD8^+^ T cells |
| BM 104 v1-v4 | % of β7^+^CD103^+^ among naïve CD8^+^ T cells |
| BM 105 v1-v4 | % of ICOS^+^ cells among Tconv cells |
| BM 106 v1-v4 | % of ICOSL^+^ cells among Tconv cells |
| BM 107 v1-v4 | % of ICOSL^+^ cells among Treg cells |
| BM 108 v1-v4 | % of ICOS^+^ cells among Treg cells |
