# Supplementary material for: Estimating causal effects of time-dependent exposures on a binary endpoint in a high-dimensional setting
Source: BMC Med Res Methodol. 2018 Jul 3;18:67. doi: 10.1186/s12874-018-0527-5 (PMC6029422; doi:10.1186/s12874-018-0527-5)
Supplement: Supplementary file 4 — Missingness graphs. It details the process used to recover missing data. (DOCX 212 kb) [file 12874_2018_527_MOESM4_ESM.docx]

**The use of missingness graph for repeated measurements of multi-dimensional biomarkers**

In studies with repeated biomarker data, missing data could be either MCAR (missing completely at random), MAR (missing at random) or MNAR (missing not at random) [1, 2]. To represent the causal mechanisms underlying in each category and the corresponding assumption about their causal impact, Mohan et al proposed the use of graphical model using conditional independencies[3, 4]. The graphical models used in this case are called missingness graphs (*m*-graphs). These graphs are an efficient way of presenting the properties of the missingness mechanisms and thus, the potential of recovering missing data. Let $G(V,E)$ be the DAG where $V$ is the set of observable nodes and $E$ the set of edges in the DAG. V can be separated into $V_{obs}$ and $V_{mis}$where $V_{obs}$ is the set of variables that are fully observed and $V_{mis}$is the set of variables that are missing in at least one record. Let $V_{i}$ a variable of interest and $V_{i}^{*}$ the variable which is actually observed, $R_{v_{i}}$ is the causal missingness mechanism of$V_{i}^{*}$. They also introduce the notion of recoverability [3] where under some conditions, an un biased estimate of given relation *Q* can be computed. If data D are generated by a process compatible with a graph G, a procedure that computes an estimator $\hat{Q}\left( D \right)$ of the relation *Q* converges to *Q* in the limit of large samples.

The reason why it is important to determine the missingness mechanism of a variable is, because missing data due to MCAR, MAR or MNAR need different approaches. For instance, MCAR data can be listwise deleted or simple imputed. For MAR, multiple imputation can provide consistent estimates while pattern mixture models seem to be most appropriate when data are MNAR [2].

Missingness mechanisms and their recoverability can be expressed in the following way:

- Missing completely at random (MCAR)

Data are called MCAR when the probability that $V_{i}$ is missing is independent from all other variables:$P\left( R_{v_{i}} | V_{obs},V_{mis} \right)=P\left( R_{v_{i}} \right)\Longleftrightarrow R_{v_{i}}\perp(V_{obs},V_{mis})$.Thus$P\left( V \right)=P\left( V|R \right)=P\left( V_{obs},V^{*} | R=0 \right)$. Since R and $V^{*}$ are currently observed, the joint distribution $P(V)$ is recoverable. Figure 1 shows an example of a MCAR model where *A* is an auxiliary variable fully observed, and *X* the variable with missing values. In this example, based on d-separation notion, the missingness mechanism $R_{x}$ is independent of all missing and fully observed variables such as X and A: $R_{x}\perp(A,X)$. The joint distribution $P(X,A)$ is then recoverable.


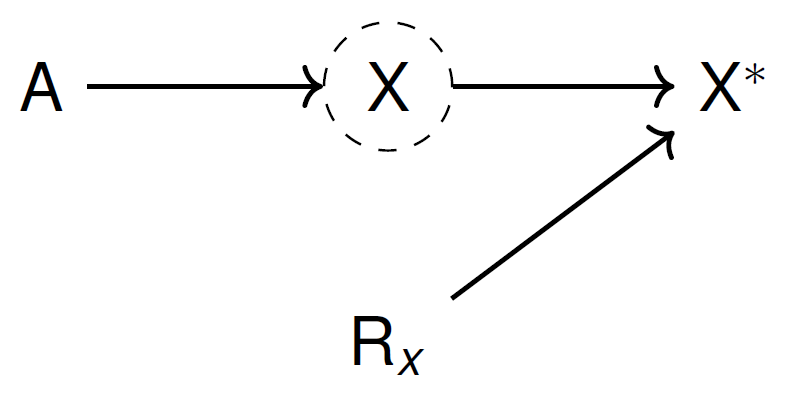


Figure 1: A simple MCAR model. Nodes with dashed circle represent variables that would have been observed had they not missing values. Nodes with a star represent observed variables with missing values.

- Missing at random (MAR)

Data are called MAR when the missingness mechanism of $V_{i}$ is conditionally independent of$V_{i}:$ $P\left( R_{v_{i}} | V_{obs},V_{mis} \right)=P\left( R_{v_{i}}|V_{obs} \right)\Longleftrightarrow R_{v_{i}}\perp V_{mis}|V_{obs}$, thus$P\left( V \right)=P\left( V_{mis}\left| V_{obs} \right)P(V_{obs} \right)= P({V^{*}|V}_{obs},R=0)P(V_{obs})$. Since R and $V^{*}$ are currently observed, the joint distribution $P(V)$ is recoverable. Figure 2 shows an example of a simple MAR model. In this example, based on the d-separation notion, the missingness mechanism $R_{x}$ and $X$ are d-connected via$A$. But, as long as $A$ is fully observed and if conditioning on$A$ blocks the path between $X$ and $R_{x}$ , then $R_{x}$ is conditionally independent of $X$ knowing$A (R_{x}\perp X|A)$. The joint distribution $P(X,A)$ is then recoverable. In this case, conditioning on a variable refers to use this variable as a predictor in a multiple imputation model [4].


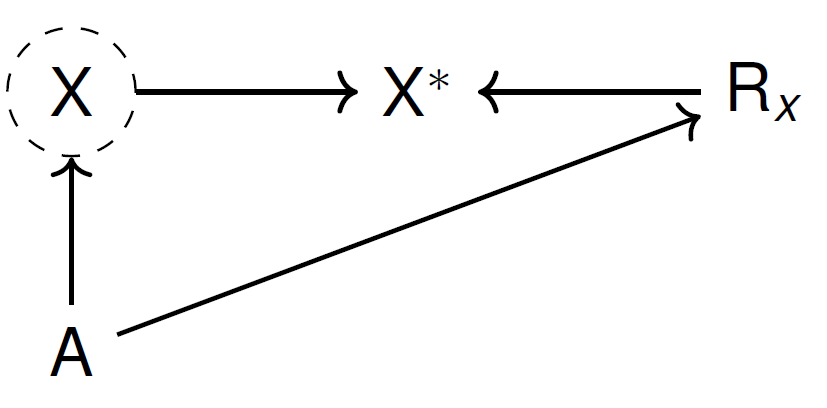


Figure 2: A simple MAR model. Nodes with dashed circle represent variables that would have been observed had they not missing values. Nodes with a star represent observed variables with missing values.

- Missing not at random (MNAR)

Data are MNAR when neither MCAR nor MAR. This can occur when the probability of a missingness mechanism is dependant of another variable:$P\left( R_{v_{i}} | V_{obs},V_{mis} \right)\neq P\left( R_{v_{i}}|V_{obs} \right)$. Figure 3 shows some typical situations where data are MNAR. Figure 3a shows the classical situation of MNAR data when there is a direct path between the missing variable and its missingness mechanism. Figure 3b shows the MNAR situation when the missing variable is d-connected to its missingness mechanism through an unobserved variable *U*. Finally in figure 3c, MNAR holds because even when conditioning on A to get *X* conditionally independent of its missingness mechanism (MAR situation), *A* is not fully observed, and hence MAR does not hold. In MNAR situation (a) and (b), *X* cannot be recovered without bias while in situation (c) *X* could be recovered with some residual bias.

**
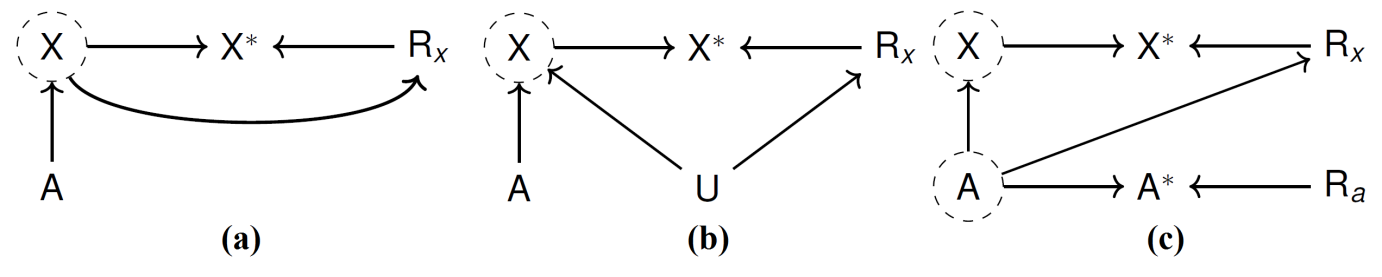
**

Figure 3: Three usual MNAR models. Missing data can be MNAR when there is a (un)directed path from a variable with missing values to its missingness mechanism (a), or through an unobserved variable U (b) or MNAR holds when all variables of a model are partially observed (c). Nodes with dashed circle represent variables that would have been observed had they not missing values. Nodes with a star represent observed variables with missing values.

The missingness graphs in figure 4 shows situations that can be found when analysing repeated immunological biomarkers. In figure 4a, $X_{i,t=1}$is d-connected to its missingness mechanism$R_{x_{i},t=1}$. However, when conditioning on$X_{i,t=0},X_{j,t=0,}X_{j,t=1}$, $X_{i,t=1}$becomes conditionally independent of its missingness mechanism${:R_{x_{i},t=1}\perp X}_{i,t=1}|X_{i,t=0}, X_{j,t=0,} X_{j,t=1.}$Thus, in this case, MAR holds and the joint distribution $P(X_{i,t=0}, X_{j,t=0,}X_{i,t=1,}X_{j,t=1} )$ is recoverable using multiple imputation with $X_{i,t=0}, X_{j,t=0,} X_{j,t=1}$ as predictor. Figure 4b illustrates a MNAR situation where a variable used to block a path (used as predictor) is partially observed (cf figure 3c). The d-separation of $X_{i,t=1}$and $X_{j,t=1}$ from their missingness mechanisms $R_{x_{i},t=1}$ and $R_{x_{j},t=1},$ requires to condition on $X_{i,t=0}, X_{j,t=0,} X_{j,t=1}$ and $X_{i,t=0}, X_{j,t=0,} X_{i,t=1}$respectively. Since in both cases, the set of nodes used to d-separate missing variables and their missingness mechanisms are not fully observed, MAR does not hold. Therefore, $X_{i,t=1}$ and $X_{j,t=1}$ can only be recovered with some residual bias in Figure 4b.


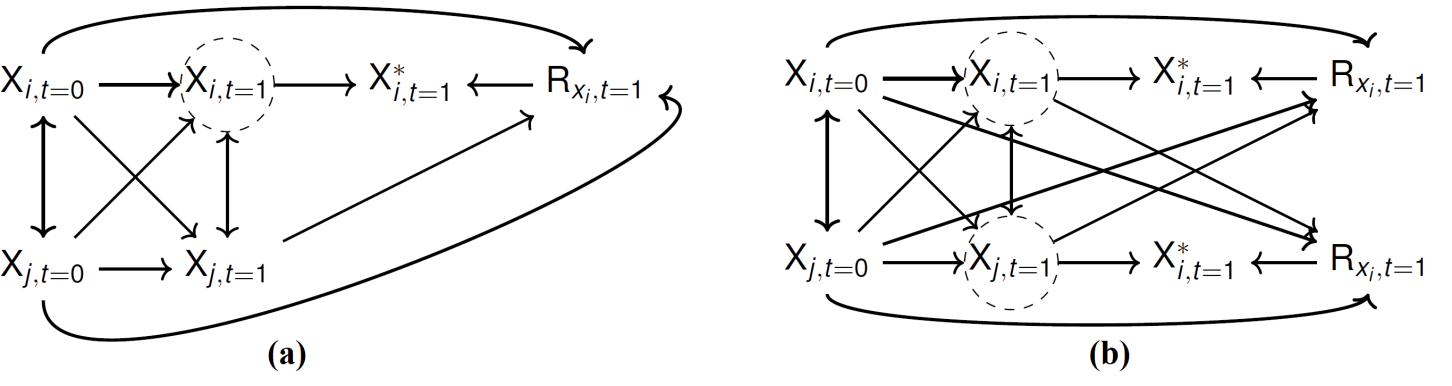


Figure 4: Different missingness mechanisms found in immunological biomarkers such as MAR (a) and MNAR (b). Nodes with dashed circle represent variables that would have been observed had they not missing values. Nodes with a star represent observed variables with missing values..

1. Rubin BYDB: **Inference and missing data**. 1976:581–592.

2. Little RA, Rubin DB: *Statistical Analysis with Missing Data*. second edi.; 2002.

3. Mohan K, Pearl J, Tian J: **Graphical Models for Inference with Missing Data**. *Nips* 2013(December):1–9.

4. Thoemmes F, Mohan K: **Graphical Representation of Missing Data Problems**. *Struct Equ Model A Multidiscip J* 2015(January):1–13.
