# Supplementary material for: Estimating causal effects of time-dependent exposures on a binary endpoint in a high-dimensional setting
Source: BMC Med Res Methodol. 2018 Jul 3;18:67. doi: 10.1186/s12874-018-0527-5 (PMC6029422; doi:10.1186/s12874-018-0527-5)
Supplement: Supplementary file 5 — Estimation of the Mean squared error. It provides supplementary tables of the simulation study results. (DOCX 27 kb) [file 12874_2018_527_MOESM5_ESM.docx]

**Estimation of the Mean squared error (MSE) of biomarkers with a true effect**

The following tables show the mean squared of the biomarkers that have a true effect on the binary outcome in all scenarios tested.

**Table 1:** Mean squared error (MSE) of biomarkers with a true effect. Parameters of the scenario were p=20, n_visits_=4, n_obs_=1000 and alpha=0.02

|  | **MSE PC-stable** | **MSE COPC-stable** |
| --- | --- | --- |
| **BM6v1** | 0.54 | 0.55 |
| **BM11v1** | 0.80 | 0.80 |
| **BM2v2** | 0.60 | 0.55 |
| **BM4v2** | 0.28 | 0.11 |
| **BM5v2** | 0.65 | 0.76 |
| **BM11v2** | 0.54 | 0.29 |
| **BM12v2** | 0.34 | 0.21 |
| **BM15v2** | 0.69 | 0.45 |
| **BM17v2** | 0.28 | 0.33 |
| **BM18v2** | 0.35 | 0.37 |
| **BM19v2** | 0.68 | 0.82 |
| **BM1v4** | 0.47 | 0.14 |
| **BM2v4** | 0.28 | 0.11 |
| **BM11v4** | 0.29 | 0.16 |
| **BM12v4** | 0.63 | 0.34 |
| **BM14v4** | 0.77 | 0.33 |

**Table 2:** Mean squared error (MSE) of biomarkers with a true effect. Parameters of the scenario were p=20, n_visits_=4, n_obs_=1000 and alpha=0.2

|  | **MSE PC-stable** | **MSE COPC-stable** |
| --- | --- | --- |
| **BM6v1** | 0.54 | 0.55 |
| **BM11v1** | 0.80 | 0.80 |
| **BM2v2** | 0.60 | 0.51 |
| **BM4v2** | 0.27 | 0.11 |
| **BM5v2** | 0.74 | 0.71 |
| **BM11v2** | 0.44 | 0.32 |
| **BM12v2** | 0.33 | 0.21 |
| **BM15v2** | 0.71 | 0.41 |
| **BM17v2** | 0.27 | 0.41 |
| **BM18v2** | 0.28 | 0.40 |
| **BM19v2** | 0.81 | 0.79 |
| **BM1v4** | 0.40 | 0.16 |
| **BM2v4** | 0.23 | 0.09 |
| **BM11v4** | 0.23 | 0.12 |
| **BM12v4** | 0.59 | 0.34 |
| **BM14v4** | 0.82 | 0.28 |

**Table 3:** Mean squared error (MSE) of biomarkers with a true effect. Parameters of the scenario were p=20, n_visits_=4, n_obs_=50 and alpha=0.02

|  | **MSE PC-stable** | **MSE COPC-stable** |
| --- | --- | --- |
| **BM6v1** | 0.54 | 0.55 |
| **BM11v1** | 0.80 | 0.80 |
| **BM2v2** | 0.60 | 0.41 |
| **BM4v2** | 0.30 | 0.06 |
| **BM5v2** | 0.81 | 0.62 |
| **BM11v2** | 0.60 | 0.47 |
| **BM12v2** | 0.36 | 0.11 |
| **BM15v2** | 0.76 | 0.36 |
| **BM17v2** | 0.42 | 0.29 |
| **BM18v2** | 0.54 | 0.32 |
| **BM19v2** | 0.98 | 0.82 |
| **BM1v4** | 0.50 | 0.09 |
| **BM2v4** | 0.38 | 0.13 |
| **BM11v4** | 0.36 | 0.07 |
| **BM12v4** | 0.70 | 0.12 |
| **BM14v4** | 0.97 | 0.21 |

**Table 4:** Mean squared error (MSE) of biomarkers with a true effect. Parameters of the scenario were p=20, n_visits_=4, n_obs_=50 and alpha=0.2

|  | **MSE PC-stable** | **MSE COPC-stable** |
| --- | --- | --- |
| **BM6v1** | 0.47 | 0.55 |
| **BM11v1** | 0.77 | 0.80 |
| **BM2v2** | 0.55 | 0.20 |
| **BM4v2** | 0.29 | 0.06 |
| **BM5v2** | 0.75 | 0.39 |
| **BM11v2** | 0.52 | 0.15 |
| **BM12v2** | 0.32 | 0.08 |
| **BM15v2** | 0.70 | 0.24 |
| **BM17v2** | 0.40 | 0.14 |
| **BM18v2** | 0.48 | 0.16 |
| **BM19v2** | 0.92 | 0.43 |
| **BM1v4** | 0.43 | 0.06 |
| **BM2v4** | 0.35 | 0.08 |
| **BM11v4** | 0.31 | 0.05 |
| **BM12v4** | 0.67 | 0.09 |
| **BM14v4** | 0.90 | 0.16 |

**Table 5:** Mean squared error (MSE) of biomarkers with a true effect. Parameters of the scenario were p=20, n_visits_=6, n_obs_=1000 and alpha=0.02

|  | **MSE PC-stable** | **MSE COPC-stable** |
| --- | --- | --- |
| **BM9v1** | 0.20 | 0.27 |
| **BM11v1** | 0.30 | 0.36 |
| **BM15v1** | 0.67 | 0.75 |
| **BM3v2** | 0.31 | 0.21 |
| **BM5v2** | 0.65 | 0.60 |
| **BM11v2** | 0.90 | 0.46 |
| **BM12v2** | 0.55 | 0.84 |
| **BM17v2** | 0.41 | 0.54 |
| **BM18v2** | 0.60 | 0.73 |
| **BM2v3** | 0.53 | 0.22 |
| **BM3v3** | 0.76 | 0.52 |
| **BM4v3** | 0.22 | 0.15 |
| **BM9v3** | 0.55 | 0.33 |
| **BM19v3** | 0.21 | 0.38 |
| **BM4v4** | 0.40 | 0.12 |
| **BM7v4** | 0.42 | 0.52 |
| **BM8v4** | 0.91 | 0.51 |
| **BM9v4** | 0.61 | 0.65 |
| **BM16v4** | 0.32 | 0.17 |
| **BM6v5** | 0.60 | 0.31 |
| **BM15v5** | 0.55 | 0.24 |
| **BM7v6** | 0.94 | 0.45 |
| **BM8v6** | 0.34 | 0.10 |

**Table 6:** Mean squared error (MSE) of biomarkers with a true effect. Parameters of the scenario were p=20, n_visits_=6, n_obs_=1000 and alpha=0.2

|  | **MSE PC-stable** | **MSE COPC-stable** |
| --- | --- | --- |
| **BM9v1** | 0.23 | 0.27 |
| **BM11v1** | 0.26 | 0.36 |
| **BM15v1** | 0.74 | 0.75 |
| **BM3v2** | 0.29 | 0.22 |
| **BM5v2** | 0.54 | 0.64 |
| **BM11v2** | 0.83 | 0.60 |
| **BM12v2** | 0.70 | 0.77 |
| **BM17v2** | 0.41 | 0.47 |
| **BM18v2** | 0.44 | 0.70 |
| **BM2v3** | 0.45 | 0.17 |
| **BM3v3** | 0.51 | 0.44 |
| **BM4v3** | 0.18 | 0.15 |
| **BM9v3** | 0.49 | 0.25 |
| **BM19v3** | 0.21 | 0.35 |
| **BM4v4** | 0.36 | 0.18 |
| **BM7v4** | 0.39 | 0.48 |
| **BM8v4** | 0.85 | 0.46 |
| **BM9v4** | 0.50 | 0.56 |
| **BM16v4** | 0.26 | 0.15 |
| **BM6v5** | 0.49 | 0.29 |
| **BM15v5** | 0.40 | 0.21 |
| **BM7v6** | 0.63 | 0.36 |
| **BM8v6** | 0.26 | 0.09 |
| **BM9v6** | 0.45 | 0.43 |

**Table 7:** Mean squared error (MSE) of biomarkers with a true effect. Parameters of the scenario were p=20, n_visits_=6, n_obs_=50 and alpha=0.02

|  | **MSE PC-stable** | **MSE COPC-stable** |
| --- | --- | --- |
| **BM9v1** | 0.26 | 0.27 |
| **BM11v1** | 0.35 | 0.36 |
| **BM15v1** | 0.75 | 0.75 |
| **BM3v2** | 0.31 | 0.29 |
| **BM5v2** | 0.71 | 0.24 |
| **BM11v2** | 0.95 | 0.47 |
| **BM12v2** | 0.93 | 0.82 |
| **BM17v2** | 0.63 | 0.58 |
| **BM18v2** | 0.74 | 0.63 |
| **BM2v3** | 0.55 | 0.34 |
| **BM3v3** | 0.77 | 0.19 |
| **BM4v3** | 0.24 | 0.15 |
| **BM9v3** | 0.60 | 0.29 |
| **BM19v3** | 0.53 | 0.22 |
| **BM4v4** | 0.43 | 0.26 |
| **BM7v4** | 0.83 | 0.39 |
| **BM8v4** | 0.96 | 0.54 |
| **BM9v4** | 0.68 | 0.36 |
| **BM16v4** | 0.37 | 0.10 |
| **BM6v5** | 0.82 | 0.22 |
| **BM15v5** | 0.73 | 0.12 |
| **BM7v6** | 0.97 | 0.25 |
| **BM8v6** | 0.36 | 0.05 |
| **BM9v6** | 0.87 | 0.26 |

**Table 8:** Mean squared error (MSE) of biomarkers with a true effect. Parameters of the scenario were p=20, n_visits_=6, n_obs_=50 and alpha=0.2

|  | **MSE PC-stable** | **MSE COPC-stable** |
| --- | --- | --- |
| **BM9v1** | 0.23 | 0.27 |
| **BM11v1** | 0.30 | 0.36 |
| **BM15v1** | 0.71 | 0.75 |
| **BM3v2** | 0.30 | 0.21 |
| **BM5v2** | 0.65 | 0.19 |
| **BM11v2** | 0.84 | 0.36 |
| **BM12v2** | 0.90 | 0.59 |
| **BM17v2** | 0.55 | 0.34 |
| **BM18v2** | 0.67 | 0.35 |
| **BM2v3** | 0.53 | 0.13 |
| **BM3v3** | 0.70 | 0.13 |
| **BM4v3** | 0.23 | 0.08 |
| **BM9v3** | 0.55 | 0.16 |
| **BM19v3** | 0.49 | 0.20 |
| **BM4v4** | 0.40 | 0.11 |
| **BM7v4** | 0.78 | 0.31 |
| **BM8v4** | 0.87 | 0.36 |
| **BM9v4** | 0.67 | 0.31 |
| **BM16v4** | 0.35 | 0.06 |
| **BM6v5** | 0.76 | 0.17 |
| **BM15v5** | 0.71 | 0.11 |
| **BM7v6** | 0.93 | 0.26 |
| **BM8v6** | 0.32 | 0.05 |
| **BM9v6** | 0.78 | 0.15 |
