# Supplementary material for: Estimating causal effects of time-dependent exposures on a binary endpoint in a high-dimensional setting
Source: BMC Med Res Methodol. 2018 Jul 3;18:67. doi: 10.1186/s12874-018-0527-5 (PMC6029422; doi:10.1186/s12874-018-0527-5)
Supplement: Supplementary file 6 — Estimation of biomarkers’ median effect. It provides supplementary tables of the application results. (DOCX 31 kb) [file 12874_2018_527_MOESM6_ESM.docx]

**Estimation of biomarkers’ median effect and PCER (per-comparison error rate)**

**Table 1:** Immunological biomarkers with a PCER < 0.5% in model 2. The number following “v” in each biomarker’s name stands for the visit number. See additional file 3 for the complete description of the biomarkers.

|  | Death | | | Progression | | | Toxicity | | |
| --- | --- | --- | --- | --- | --- | --- | --- | --- | --- |
| Rank | **Biomarker** | **Median effect** | **PCER** | **Biomarker** | **Median effect** | **PCER** | **Biomarker** | **Median effect** | **PCER** |
| 1 | BM16v2 | 0.81 | 0.0035 | BM8v1 | 0.77 | 0.0031 | BM7v4 | 0.79 | 0.0028 |
| 2 | BM5v1 | 0.81 | 0.0035 | BM44v4 | 0.72 | 0.0036 | BM8v4 | 0.76 | 0.0034 |
| 3 | BM42v3 | 0.80 | 0.0037 | BM26v2 | 0.71 | 0.0041 | BM16v3 | 0.75 | 0.0036 |
| 4 | BM48v1 | 0.86 | 0.0037 | BM30v3 | 0.71 | 0.0041 | BM26v4 | 0.75 | 0.0036 |
| 5 | BM42v2 | 0.80 | 0.0038 | BM44v3 | 0.68 | 0.0042 | BM7v3 | 0.76 | 0.0037 |
| 6 | BM14v4 | 0.79 | 0.0039 | BM45v1 | 0.70 | 0.0047 | BM9v4 | 0.72 | 0.0039 |
| 7 | BM30v4 | 0.80 | 0.0039 | BM39v4 | 0.66 | 0.0049 | BM39v3 | 0.72 | 0.0039 |
| 8 | BM11v4 | 0.83 | 0.0040 | BM40v4 | 0.66 | 0.0049 | BM32v3 | 0.71 | 0.0042 |
| 9 | BM11v1 | 0.76 | 0.0042 | BM14v2 | 0.66 | 0.0050 | BM30v1 | 0.67 | 0.0045 |
| 10 | BM9v4 | 0.81 | 0.0043 |  |  |  | BM45v1 | 0.71 | 0.0046 |
| 11 | BM17v1 | 0.77 | 0.0043 |  |  |  | BM18v3 | 0.71 | 0.0048 |
| 12 | BM11v2 | 0.78 | 0.0045 |  |  |  |  |  |  |
| 13 | BM30v1 | 0.76 | 0.0046 |  |  |  |  |  |  |
| 14 | BM31v3 | 0.75 | 0.0047 |  |  |  |  |  |  |
| 15 | BM48v3 | 0.78 | 0.0048 |  |  |  |  |  |  |
| 16 | BM25v2 | 0.77 | 0.0049 |  |  |  |  |  |  |
| 17 | BM10v4 | 0.77 | 0.0050 |  |  |  |  |  |  |

**Table 2:** Immunological biomarkers with a PCER < 0.5% in model 3. The number following “v” in each biomarker’s name stands for the visit number. See additional file 3 for the complete description of the biomarkers.

|  | Death | | | Progression | | | Toxicity | | |
| --- | --- | --- | --- | --- | --- | --- | --- | --- | --- |
| Rank | **Biomarker** | **Median effect** | **PCER** | **Biomarker** | **Median effect** | **PCER** | **Biomarker** | **Median effect** | **PCER** |
| 1 | BM60v3 | 0.87 | 0.0015 | BM78v4 | 0.74 | 0.0024 | BM96v4 | 0.86 | 0.0013 |
| 2 | BM87v1 | 0.83 | 0.0021 | BM58v2 | 0.74 | 0.0025 | BM87v1 | 0.81 | 0.0018 |
| 3 | BM96v4 | 0.84 | 0.0021 | BM108v4 | 0.72 | 0.0025 | BM8v4 | 0.80 | 0.0019 |
| 4 | BM105v4 | 0.83 | 0.0022 | BM105v4 | 0.73 | 0.0026 | BM9v4 | 0.72 | 0.0026 |
| 5 | BM58v2 | 0.81 | 0.0025 | BM8v1 | 0.72 | 0.0028 | BM7v4 | 0.76 | 0.0029 |
| 6 | BM58v4 | 0.80 | 0.0026 | BM108v3 | 0.68 | 0.0031 | BM57v3 | 0.75 | 0.0029 |
| 7 | BM11v4 | 0.81 | 0.0026 | BM86v4 | 0.63 | 0.0031 | BM88v3 | 0.69 | 0.0032 |
| 8 | BM76v3 | 0.80 | 0.0027 | BM80v1 | 0.71 | 0.0031 | BM59v1 | 0.76 | 0.0032 |
| 9 | BM108v4 | 0.80 | 0.0027 | BM59v4 | 0.71 | 0.0032 | BM62v3 | 0.72 | 0.0033 |
| 10 | BM11v1 | 0.78 | 0.0027 | BM89v3 | 0.67 | 0.0032 | BM93v3 | 0.72 | 0.0034 |
| 11 | BM9v4 | 0.80 | 0.0028 | BM65v4 | 0.69 | 0.0032 | BM65v3 | 0.69 | 0.0034 |
| 12 | BM68v4 | 0.77 | 0.0028 | BM66v4 | 0.68 | 0.0032 | BM89v3 | 0.72 | 0.0034 |
| 13 | BM98v4 | 0.78 | 0.0028 | BM56v3 | 0.68 | 0.0033 | BM7v3 | 0.72 | 0.0036 |
| 14 | BM52v1 | 0.78 | 0.0028 | BM86v3 | 0.68 | 0.0033 | BM63v1 | 0.67 | 0.0037 |
| 15 | BM87v2 | 0.81 | 0.0028 | BM63v1 | 0.68 | 0.0034 | BM69v3 | 0.65 | 0.0038 |
| 16 | BM63v4 | 0.77 | 0.0029 | BM67v3 | 0.69 | 0.0035 | BM11v2 | 0.68 | 0.0040 |
| 17 | BM74v4 | 0.77 | 0.0029 | BM70v3 | 0.68 | 0.0036 | BM104v3 | 0.62 | 0.0040 |
| 18 | BM11v2 | 0.78 | 0.0030 | BM83v3 | 0.65 | 0.0039 | BM6v2 | 0.67 | 0.0041 |
| 19 | BM59v1 | 0.76 | 0.0031 | BM9v2 | 0.63 | 0.0039 | BM80v3 | 0.69 | 0.0043 |
| 20 | BM63v3 | 0.77 | 0.0032 | BM56v4 | 0.65 | 0.0041 | BM103v3 | 0.66 | 0.0043 |
| 21 | BM65v3 | 0.75 | 0.0033 | BM96v2 | 0.62 | 0.0043 | BM10v2 | 0.64 | 0.0043 |
| 22 | BM100v3 | 0.76 | 0.0033 | BM61v3 | 0.63 | 0.0044 | BM98v2 | 0.65 | 0.0046 |
| 23 | BM10v4 | 0.77 | 0.0033 | BM60v4 | 0.63 | 0.0045 | BM96v3 | 0.69 | 0.0046 |
| 24 | BM101v3 | 0.75 | 0.0034 | BM98v1 | 0.61 | 0.0046 | BM83v3 | 0.65 | 0.0046 |
| 25 | BM87v3 | 0.69 | 0.0036 | BM101v3 | 0.65 | 0.0046 | BM75v3 | 0.45 | 0.0047 |
| 26 | BM105v2 | 0.71 | 0.0037 | BM56v2 | 0.64 | 0.0047 | BM91v2 | 0.60 | 0.0047 |
| 27 | BM5v1 | 0.76 | 0.0037 | BM98v2 | 0.61 | 0.0047 | BM72v3 | 0.62 | 0.0048 |
| 28 | BM106v4 | 0.75 | 0.0038 | BM10v3 | 0.61 | 0.0047 | BM90v3 | 0.60 | 0.0049 |
| 29 | BM6v3 | 0.70 | 0.0039 | BM55v4 | 0.59 | 0.0048 | BM56v2 | 0.65 | 0.0049 |
| 30 | BM57v3 | 0.69 | 0.0039 | BM93v4 | 0.62 | 0.0050 | BM9v3 | 0.64 | 0.0050 |
| 31 | BM54v4 | 0.71 | 0.0040 | BM100v4 | 0.65 | 0.0050 |  |  |  |
| 32 | BM7v2 | 0.72 | 0.0040 | BM85v2 | 0.61 | 0.0050 |  |  |  |
| 33 | BM79v3 | 0.71 | 0.0040 |  |  |  |  |  |  |
| 34 | BM8v2 | 0.63 | 0.0043 |  |  |  |  |  |  |
| 35 | BM70v3 | 0.69 | 0.0045 |  |  |  |  |  |  |
| 36 | BM61v2 | 0.74 | 0.0047 |  |  |  |  |  |  |
| 37 | BM65v4 | 0.65 | 0.0048 |  |  |  |  |  |  |
| 38 | BM78v4 | 0.75 | 0.0050 |  |  |  |  |  |  |
| 39 | BM63v1 | 0.71 | 0.0050 |  |  |  |  |  |  |
